# Restricting Dosage Compensation Complex Binding to the X Chromosomes by H2A.Z/HTZ-1

**DOI:** 10.1371/journal.pgen.1000699

**Published:** 2009-10-23

**Authors:** Emily L. Petty, Karishma S. Collette, Alysse J. Cohen, Martha J. Snyder, Györgyi Csankovszki

**Affiliations:** Department of Molecular, Cellular, and Developmental Biology, University of Michigan, Ann Arbor, Michigan, United States of America; Massachusetts General Hospital, Howard Hughes Medical Institute, United States of America

## Abstract

Dosage compensation ensures similar levels of X-linked gene products in males (XY or XO) and females (XX), despite their different numbers of X chromosomes. In mammals, flies, and worms, dosage compensation is mediated by a specialized machinery that localizes to one or both of the X chromosomes in one sex resulting in a change in gene expression from the affected X chromosome(s). In mammals and flies, dosage compensation is associated with specific histone posttranslational modifications and replacement with variant histones. Until now, no specific histone modifications or histone variants have been implicated in *Caenorhabditis elegans* dosage compensation. Taking a candidate approach, we have looked at specific histone modifications and variants on the *C. elegans* dosage compensated X chromosomes. Using RNAi-based assays, we show that reducing levels of the histone H2A variant, H2A.Z (HTZ-1 in *C. elegans*), leads to partial disruption of dosage compensation. By immunofluorescence, we have observed that HTZ-1 is under-represented on the dosage compensated X chromosomes, but not on the non-dosage compensated male X chromosome. We find that reduction of HTZ-1 levels by RNA interference (RNAi) and mutation results in only a very modest change in dosage compensation complex protein levels. However, in these animals, the X chromosome–specific localization of the complex is partially disrupted, with some nuclei displaying DCC localization beyond the X chromosome territory. We propose a model in which HTZ-1, directly or indirectly, serves to restrict the dosage compensation complex to the X chromosome by acting as or regulating the activity of an autosomal repellant.

## Author Summary

In organisms where females have two X chromosomes and males only have one, a mechanism called dosage compensation ensures that both sexes receive the same amount of information from their X chromosomes. Disruption of dosage compensation leads to lethality in the affected sex. While the precise mechanisms of dosage compensation differ between organisms, changes to the structure of the X chromosomes are involved in each case. The DNA of all chromosomes is packaged into a complex protein–DNA structure called chromatin. The most basic level of packaging involves wrapping DNA around a group of small proteins called histones. In both mammals and flies, dosage compensation is associated with specific changes to the histones on the dosage compensated X chromosome. Until now, no such change has been associated with dosage compensation in worms. Here we present evidence that the histone variant HTZ-1/H2A.Z plays a role in dosage compensation in the worm. Specifically, we suggest that HTZ-1 functions to ensure that only the X chromosomes, and not the other chromosomes, are subjected to dosage compensation. This suggests that, despite different mechanisms, one common theme of dosage compensation is a change at the level of the histones associated with the chromosomal DNA.

## Introduction

Many species- such as humans, mice, flies, and worms- utilize a chromosome-based mechanism to establish sex. This results in a difference in sex chromosome number between the sexes that, left uncorrected, puts one sex at a great selective disadvantage. In order to combat this, these organisms employ a second mechanism to ensure that the same amount of sex chromosome-linked gene expression occurs in both sexes. This mechanism is called dosage compensation [1]–[4].

In flies and mammals, specific posttranslational histone modifications and/or replacement of core histones with variants are key features of the dosage compensated X chromosomes [5]. Dosage compensation in flies is brought about by the MSL (*m*ale-*s*pecific-*l*ethal) complex that localizes to the single X chromosome in males resulting in a two-fold increase in gene expression [6],[7]. The MSL complex is made up of at least five proteins (MSL1 [8], MSL2 [9], MSL3 [10], MLE [11], and MOF [12]), and one of two non-coding RNAs (roX 1 and roX2, *R*NA *o*n the *X* [13],[14]). The hypertranscribed male X is enriched for histone H4 lysine 16 acetylation (H4K16ac) [15]. MOF (*m*ales-absent *o*n the *f*irst) places the H4K16ac mark on the male X and this function is essential for dosage compensation [16]–[18]. In mammals, one of the two female X chromosomes is transcriptionally inactivated [3]. The inactive X is targeted for silencing by the non-coding RNA, Xist in mice [19],[20], XIST in humans [21], that coats the inactive X chromosome [22]. This is followed by chromosome-wide histone H3 lysine 27 tri-methylation (H3K27me3) by Polycomb repressor complex 2 (PRC2) [23], and histone H2A and H2A.Z mono-ubiquitylation by Polycomb repressor complex 1 (PRC1) [24],[25]. On the inactive X chromosome, there is also an enrichment of histone H3 lysine 9 dimethylation (H3K9me2) [26] and an enrichment of the histone variant macroH2A [27]. However, other modifications and variants are specifically under-represented on the inactive X: di-, and trimethylation of histone H3 lysine 4 (H3K4me) [26],[28], dimethylation of histone H3 arginine 17 (H3R17me2) and H3 lysine 36 (H3K36me2) [29], acetylation of the N-terminal tails of histones H2A, H3 and H4 [30]–[32], and the phosphorylated form of macroH2A1 [33]. These and other modifications are thought to be vital for the resulting essential change in X-linked gene expression in male flies and female mammals.

In *C. elegans*, dosage compensation is achieved by the dosage compensation complex (DCC), which binds both X chromosomes in hermaphrodites to downregulate gene expression two-fold [2]. DPY-27 [34], MIX-1 [35], DPY-26 [36], DPY-28 [37], and CAPG-1 [38] form a condensin-like complex, condensin I^DC^. SDC-2 [39], SDC-3 [40], and DPY-30 [41] are thought to be responsible for recruitment of the condensin-like complex, as well as DPY-21 [39] and SDC-1 [42],[43], to the X chromosomes in hermaphrodites. All DCC proteins, except for SDC-2, are supplied maternally to the oocyte, and are initially present in both male and hermaphrodite embryos [2]. SDC-2 is not contributed maternally, expressed only in hermaphrodites and is thought to confer both sex-specificity and X-chromosome specificity to dosage compensation [39]. The DCC initially binds to *rex* (*r*ecruitment *e*lements on the *X*) sites, which represent sites of DCC enrichment on the X chromosome, and spreads in *cis* along the lengths of both X chromosomes in the hermaphrodite [44]–[47]. As a result, gene expression from the two hermaphrodite X chromosomes is down-regulated by half, thus limiting X-linked gene products to levels produced in XO males [48]. Condensin complexes are well known for their roles in affecting chromosome architecture during mitosis and meiosis [49], so it is believed that the DCC may be altering the overall organization of the X chromosomes to dampen gene expression during interphase. A chromosome-wide architectural change by the DCC condensin may require or lead to specific modifications to the basic organizational unit of chromatin, the nucleosome. However, no nucleosomal changes, such as posttranslational modification of histones or histone variants, have been previously implicated to play a role in *C. elegans* dosage compensation.

While in somatic cells of hermaphrodites the X chromosome is subject to dosage compensation, in the postembryonic germ line of both sexes the X is subject to a distinct form of chromosome-wide regulatory process: global repression throughout meiosis in males and during early meiosis in hermaphrodites [50]. The *m*aternal *e*ffect *s*terility (MES) proteins mediate silencing of the germ line X chromosome and their function is required for germ line viability [50]–[52]. Three of the *mes* genes (*mes-2*, *mes-3*, and *mes-6*) encode proteins that function together in a PRC2-like complex, which localizes to the germ line X-chromosome(s) and leads to enrichment of H3 lysine 27 trimethylation on the X [53]–[56]. By contrast, an additional MES protein, MES-4, localizes only to the autosomes and not X, and its function is necessary for germ line X silencing [51],[57]. Additionally, the silenced germ line X chromosomes show a significant depletion of activating marks such as acetylation of the N terminal tail of histone H4 and methylation of lysine 4 on H3 [50],[58].

From studies of dosage compensation in other organisms and of germ line X chromosome silencing in *C. elegans*, there are many well-documented links between different forms of chromosome-wide gene regulation and specific nucleosome characteristics. This led us to explore whether we might find a similar link between *C. elegans* dosage compensation and nucleosome composition. We were interested to see if any histone modifications or histone variants play a functional role in dosage compensation in worms. In this paper we report on the role of the *C. elegans* histone H2A.Z variant (HTZ-1).

The histone variant H2A.Z is conserved from yeast to humans and has been implicated in diverse biological processes. Interestingly, depending on its histone partner in the nucleosome core particle, H2A.Z can either stabilize or destabilize the nucleosome [59]. When partnered with histone H3, the H2A.Z-containing nucleosome becomes more stable, but when partnered with the histone variant H3.3, the nucleosome becomes destabilized. Unstable H2A.Z/H3.3. nucleosomes may function to poise genes for activation. Consistently, studies in several organisms implicate H2A.Z in various aspects of transcription activation. In *Tetrahymena*, hv1/H2A.Z associates with the transcriptionally active macronucleus [60]–[62]. Genome-wide localization studies in yeast [63]–[66], worms [67], flies [68], plants [69], and humans [70], revealed that H2A.Z preferentially localizes to 5′ ends of genes, consistent with a role in transcription activation. Loss of Htz1 has been shown to diminish RNA Pol II binding to promoters, slow the activation of regulated genes, or prevent rapid reactivation of recently repressed genes [66],[71],[72]. A role of HTZ-1 to poise genes for rapid activation has also been observed in a study of the *C. elegans* H2A.Z homolog, HTZ-1 [73].

However, H2A.Z also localizes to regulatory regions not corresponding to promoters to exert other functions. In budding yeast, Htz1 also functions at boundary elements to protect genes from heterochromatinization by antagonizing the spread of silencing complexes [74]. This antisilencing functions at the global level, not just locally [75]. Consistent with an antisilencing role, in plants, H2A.Z antagonizes DNA methylation [69]. H2A.Z also localizes to insulator elements in chicken [76], and to functional regulatory elements in human cells [70]. It has been proposed that in this context, the presence of an H2A.Z/H3.3 labile nucleosome prevents the spreading of heterochromatic marks [59].

On the other hand, H2A.Z also plays a role in heterochromatin formation. In this context, H2A.Z most likely partners with H3 to form stable nucleosomes [59]. In mammals and in flies, H2A.Z associates with pericentric heterochromatin and interacts with heterochromatin protein HP1 [77]–[80]. Mammalian H2A.Z also becomes incorporated into the inactive XY body following meiosis [81]. However, H2A.Z is significantly underrepresented and differentially modified on the mammalian inactive X chromosome in somatic cells, indicating that H2A.Z enrichment is not a general feature of all heterochromatin [25],[79],[82]. Consistent with that, H2A.Z is not enriched at heterochromatic chromocenters in plants [69],[83].

Here we show that in *C. elegans* the histone variant H2A.Z/HTZ-1 functions in dosage compensation. Consistent with previous reports [67], we find that HTZ-1 is under-represented on the dosage compensated X chromosomes in somatic nuclei of hermaphrodites. However, we do not observe HTZ-1 depletion on the non-dosage compensated X chromosome in male somatic nuclei. We also see an underrepresentation of HTZ-1 on the silent X chromosomes of both male and hermaphrodite germ nuclei. Partial depletion of HTZ-1 does not lead to an overall decrease in DCC protein levels. Instead we see mislocalization of the DCC away from the X chromosomes and onto autosomes. These results reveal an HTZ-1-dependent activity that serves to repel the DCC away from autosomes. We propose that HTZ-1 plays a role in dosage compensation by directly or indirectly restricting binding of the DCC to the X chromosomes.

## Results

### HTZ-1 function promotes dosage compensation

To search for chromatin modifiers involved in worm dosage compensation, we utilized two RNAi-based assays in a genetic background sensitized for detecting disturbances in dosage compensation. We tested genes encoding *C. elegans* homologs of histone variants, genes implicated in modifying chromatin via posttranslational histone modifications (such as acetylation or methylation) or chromatin remodeling [84], as well as genes annotated to contain chromo-, bromo- or SET domains (Wormbase [http://www.wormbase.org], release WS201).

The first assay was completed in the *sex-1(y263)* mutant background. *sex-1* functions genetically as an X signal element by repressing *xol-1*, the master switch regulating both sex-determination and dosage compensation [85],[86]. In addition, *sex-1* plays a role downstream of *xol-1*, promoting dosage compensation in hermaphrodites [87]. In *sex-1(y263)* mutant hermaphrodites, dosage compensation is partially impaired, resulting in 15–30% embryonic lethality. In these worms, partial loss-of-function due to feeding RNAi of a gene important for dosage compensation leads to increased lethality [87]. A second genetic assay was based on the rescue of males that inappropriately turn on dosage compensation due to a *xol-1(y9)* mutation. Expression of *xol-1* in males is essential to prevent dosage compensation of the single X chromosome [88]. Mutations in *xol-1* are male lethal due to ectopic dosage compensation, leading to abnormally low levels of X-linked gene expression. The *sex-1(y263)* mutation partially weakens dosage compensation, as described above. *xol-1(y9) sex-1(y263)* males die, but they can be rescued by feeding RNAi of dosage compensation genes [38],[87]. To ensure a consistent proportion of males in our test strain, we perform these assays in a strain that also carries the *him-8(e1489)* allele. Mutations in *him-8* cause X chromosome nondisjunction in meiosis and results in a predictable 38% of XO progeny each generation [89].

**Figure 1:**
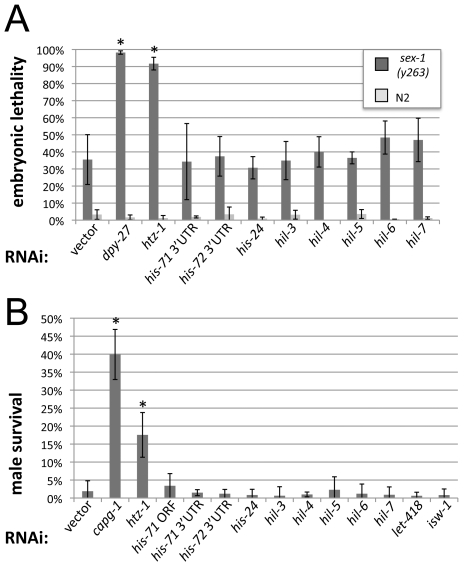
HTZ-1 function is needed for dosage compensation. (A) Embryonic lethality caused by feeding RNAi in wild type (N2) and *sex-1(y263)* X hermaphrodite worms. RNAi of both *dpy-27* (n_N2_ = 1173; n*_sex-1_* = 566) and *htz-1* (n_N2_ = 1292; n*_sex-1_* = 385) leads to synergistic embryonic lethality in the *sex-1* mutant background that is significantly different from levels observed after vector RNAi (n_N2_ = 459; n*_sex-1_* = 504) (p = 1.84×10^−5^, and p = 4.9×10^−5^ respectively). RNAi of *his-71* (3′-UTR, n_N2_ = 1901 ;n*_sex-1_* = 1037), *his-72* (3′-UTR, n_N2_ = 2821; n*_sex-1_* = 1381), *his-24* (n_N2_ = 438; n*_sex-1_* = 933), *hil-3* (n_N2_ = 378; n*_sex-1_* = 682), *hil-4* (n_N2_ = 412; n*_sex-1_* = 853), *hil-5* (n_N2_ = 440; n*_sex-1_* = 1083), *hil-6* (n_N2_ = 350; n*_sex-1_* = 481 ), or *hil-7* (n_N2_ = 413; n*_sex-1_* = 882) does not significantly affect *sex-1* embryonic lethality compared to vector. (B) Male survival caused by feeding RNAi in *him-8(e1489) IV*; *xol-1(y9) sex-1(y263)* X worms. Both *capg-1* (n = 771) and *htz-1* (n = 1337) RNAi rescue a significant proportion of males (p = 5. 2×10^−6^ and p = 3.8×10^−4^ respectively) compared to vector (n = 1245). RNAi of *his-71* (coding region, n = 623), *his-71* (3′-UTR, n = 1786), *his-72* (3′-UTR, n = 639), *his-24* (n = 633), *hil-3* (n = 810), *hil-4* (n = 1332), *hil-5* (n = 1847), *hil-6* (n = 1139), and *hil-7* (n = 868), *isw-1*(n = 1004), or *let-418* (n = 1313) does not lead to significant male rescue. Error bars indicate standard deviation for four experiments. Asterisks indicate a p value of less than 0.05 by student's T-test analysis comparing vector and experimental RNAi data.

RNAi of DCC components show near complete *sex-1* lethality and results in 33–60% rescue of *him-8(e1489)*; *xol-1(y9) sex-1(y263)* males in these two assays [38]. One candidate, the histone variant *htz-1* (*C. elegans* H2A.Z homolog) showed a similar genetic interaction. RNAi in the wild type background leads to little to no lethality, while *htz-1* RNAi in the *sex-1(y263)* background leads to near complete embryonic lethality (Figure 1A). In the *him-8(e1489)*; *xol-1(y9) sex-1(y263)* background, RNAi of the histone variant *htz-1* resulted in over 15% rescue (Figure 1B). To ensure that these phenotypes are not caused by general disruption to chromatin, we also tested two genes encoding H3.3 histone variants (*his-71* and *his-72*) [90], and genes encoding linker histones [*his-24* (H1.1), *hil-3* (H1.3), *hil-4* (H1.4), *hil-5* (H1.5), *hil-6* (H1.6), and *hil-7* (H1.Q)] [91]. RNAi of these genes did not show similar genetic interactions. RNAi of many other chromatin factors also failed to result in significant male rescue (for a complete list, see Table S1). The chromatin remodeling enzyme *isw-1*, and the histone deacetylase *let-418* are shown as examples (Figure 1B). We conclude that depletion of HTZ-1 leads to disruption of dosage compensation.

### HTZ-1 is underrepresented on hermaphrodite X chromosomes but not the male X chromosome

A previous study found that in worms HTZ-1 preferentially localizes to promoters, as in other organisms [67]. Furthermore, fewer peaks of HTZ-1 incorporation were found on the X chromosome, as compared to autosomes. The authors attribute this difference to the relative lack of developmentally important genes on the X chromosome, rather than a direct role in dosage compensation [67]. Our RNAi data above indicates that HTZ-1 function is needed for wild type levels of dosage compensation, but does not address whether this role is direct or indirect. That is, *htz-1* may directly regulate some aspect of DCC function, or *htz-1* may indirectly affect dosage compensation by regulating expression of known or unknown dosage compensation genes. To begin to distinguish between these possibilities, we analyzed the distribution of HTZ-1 in male and hermaphrodite nuclei. We reasoned that if HTZ-1 functions in dosage compensation, its distribution in the nucleus may be different in males (dosage compensation inactive) and hermaphrodites (dosage compensation active).

**Figure 2:**
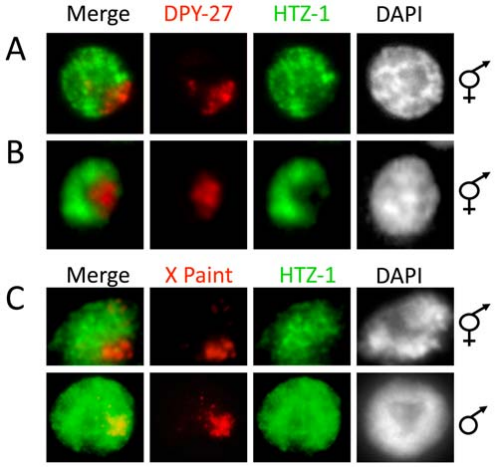
HTZ-1 depletion on dosage compensated X chromosomes. HTZ-1 and DPY-27 localization by IF in hermaphrodite adult somatic nucleus (A) and embryonic nucleus after the onset of dosage compensation (B). HTZ-1 (green) staining is reduced in the region containing the X chromosomes, as marked by DPY-27 (red) staining. (C) HTZ-1 localization by IF (green) and X-Chromosome labeling by FISH (red) in adult hermaphrodite (top) and male (bottom) somatic nuclei. HTZ-1 staining is reduced on the hermaphrodite X chromosomes but not on the male X chromosome.

To analyze HTZ-1 distribution, we took advantage of a strain expressing a YFP-HTZ-1 fusion protein, or used an HTZ-1 specific antibody. The specificity of our HTZ-1 antibody is demonstrated by recognition of a protein of the predicted size on western blots and reduction of signal after HTZ-1 depletion on both western blots and by immunofluorescence (IF) (Figure S1). We marked the X-chromosome territory with an antibody specific to DPY-27 (marks the X chromosomes in hermaphrodites only) or X-paint fluorescent *in situ* hybridization (FISH) (to mark the X chromosomes in both sexes). Consistent with a previous report [67], we observed reduced HTZ-1 staining on the dosage compensated X chromosomes in mid-to-late stage hermaphrodite embryos after the onset of dosage compensation by DPY-27/HTZ-1 IF (Figure 2A and 2B), and combined X-Paint FISH/HTZ-1 IF (Figure 2C). We also observed reduced levels of YFP-HTZ-1 in the territory of the X-chromosomes in transgenic hermaphrodite embryos (Figure S2). However, in males we did not observe a decrease in HTZ-1 staining intensity in the X chromosome territory of somatic nuclei (Figure 2C). These results indicate that reduced HTZ-1 levels are specific to dosage compensated X chromosomes and not a general feature of X chromosomes in both sexes in adult animals.

### HTZ-1 depletion does not lead to a decrease in DCC protein levels

**Figure 3:**
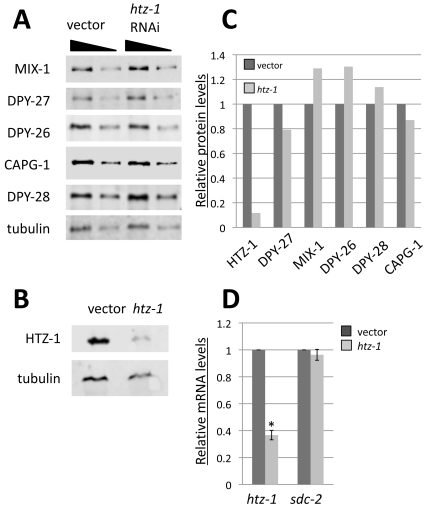
*htz-1* RNAi does not significantly decrease DCC levels. HTZ-1 levels were reduced by feeding RNAi in wild type worms. (A,B) An equal number of control vector and *htz-1* RNAi adult animals were collected for quantitative western blot analysis to observe levels of HTZ-1, MIX-1, DPY-27, DPY-26, CAPG-1, DPY-28 and α-Tubulin (loading control) after RNAi treatment. Band intensities were quantified and normalized to tubulin (C). RNAi significantly reduces HTZ-1 levels, but no significant decrease was seen in levels of DCC subunits. Vector and *htz-1* RNAi adults were also collected for RT-qPCR analysis (D). *htz-1* expression is significantly reduced in *htz-1* animals (p = 3.1×10^−6^), but there is no significant difference in *sdc-2* expression between vector and *htz-1* RNAi animals.

The results of the genetic assays and localization assays appeared contradictory: reduced *htz-1* expression disrupts dosage compensation, yet the protein itself is depleted on the dosage compensated X chromosomes. Therefore, we wanted to explore how dosage compensation is affected in *htz-1* depleted animals. If HTZ-1 functions in dosage compensation indirectly (by regulating expression of dosage compensation genes) we would predict to see a decrease in DCC protein levels upon HTZ-1 depletion. We analyzed worms carrying the *htz-1* deletion allele *tm2469* that removes 345 of 885 base pairs from *htz-1* and likely represents a null allele. *htz-1(tm2469)* homozygous progeny of heterozygous mothers (m^+^z^−^) develop into healthy adults but are sterile, as reported [67]. However, the *tm2469* deletion appears to affect expression of not just *htz-1*, but the neighboring gene as well (Figure S3). It was therefore important to obtain HTZ-1-depleted worms using an alternate method and to confirm that phenotypes are due to HTZ-1 depletion, and not depletion of the neighboring gene product. As an alternative method, we depleted HTZ-1 levels by feeding worms bacteria expressing double stranded RNA corresponding to *htz-1*. As a control, worms were fed bacteria carrying an empty vector. Feeding RNAi in wild type animals greatly reduced HTZ-1 as detected both by immunofluorescence and quantitative Western blot analyses (89% reduction) (Figure 3B and Figure S1).

To investigate the possibility that HTZ-1 depletion leads to a decrease in DCC protein levels, we quantified protein levels by western blotting of HTZ-1 depleted and control animals. Although HTZ-1 levels were clearly reduced after *htz-1* RNAi, we did not observe a dramatic change in DCC protein levels (Figure 3A and 3C). Levels of DPY-27 and CAPG-1 show a very slight decrease while MIX-1, DPY-26, and DPY-28 show very slight increases after *htz-1* RNAi. Our results suggest that, HTZ-1 reduction does not lead to a significant defect in overall DCC protein levels. However, we cannot exclude the possibility that the timing of DCC gene expression is changed (delayed) in HTZ-1 depleted cells, as was observed for genes involved in foregut development [73]. It is also possible that a small amount HTZ-1 that remains after feeding RNAi is sufficient for DCC gene expression, but more complete HTZ-1 depletion would result in a significant decrease in DCC protein levels.

SDC-2, the primary determinant of hermaphrodite fate, is the only DCC protein whose expression in the zygote is essential [39]. The remaining DCC proteins are maternally loaded into the oocyte and this maternal load is sufficient to carry out dosage compensation in the developing embryo. Therefore, it was important to determine whether *sdc-2* transcript levels are affected after HTZ-1 depletion. We analyzed *sdc-2* mRNA levels in HTZ-1 depleted and control animals by reverse transcription followed by quantitative polymerase chain reaction (RT-qPCR) and observed no significant change in *sdc-2* expression (Figure 3D). We conclude that the changes observed in DCC protein and RNA levels are not likely to be sufficient to explain the observed requirement for HTZ-1 to maintain wild type levels of dosage compensation.

### DCC localization is disrupted in HTZ-1-depleted animals

**Figure 4:**
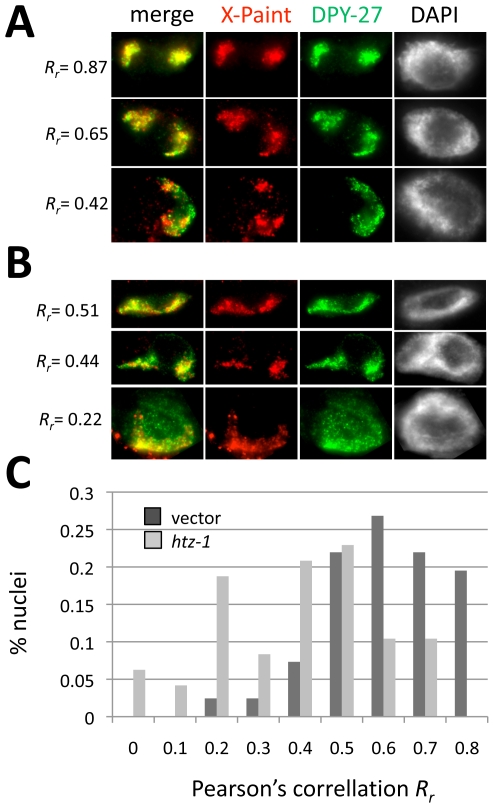
HTZ-1 depletion disrupts DCC restriction to the X chromosomes. Adult hermaphrodite intestinal nuclei were stained with α-DPY-27 (green), X-paint FISH probe (red) and DAPI (gray). (A) Representative nuclei observed after vector RNAi treatment. (B) Representative nuclei observed after *htz-1* RNAi treatment. (C) Summary of quantification of DPY-27 colocalization with the X chromosomes following vector and *htz-1* RNAi treatment. After vector RNAi, the average *R_r_* value from three independent experiments was 0.65±0.14 (n = 41). After *htz-1* RNAi, the average value from three independent experiments was reduced to 0.44±0.2 (n = 48).

An alternative possibility is that HTZ-1 has a more direct role in dosage compensation by affecting DCC localization or function. To explore this possibility, we used immunofluorescence to observe DCC localization in HTZ-1-depleted worms. The DCC was clearly present in nuclei of *htz-1(RNAi)* animals, again suggesting that HTZ-1 depletion does not lead to a significant reduction in DCC protein levels. However, the territory occupied by the DCC in these nuclei was significantly more diffuse in appearance than in wild type nuclei (Figure 4).

**Figure 5:**
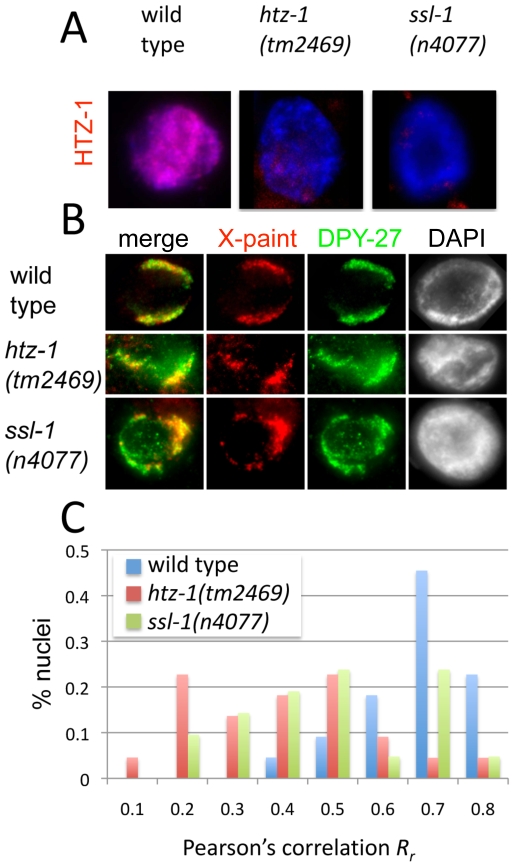
DCC mislocalization in *htz-1(tm2469)* and *ssl-1(n4077)* adult hermaphrodites. (A) HTZ-1 (red) and DAPI (blue) staining in wild-type, *htz-1(tm2469)* m+z− and *ssl-1(n4077)* m+z− adult hermaphrodite intestinal nuclei. HTZ-1 levels are reduced in *htz-1(tm2469)*m+z− and *ssl-1(n4077)* m+z− adult hermaphrodites. (B) Representative wild type (*R_r_* = 0.67), *htz-1(tm2469)*m+z− (*R_r_* = 0.18) and *ssl-1(n4077)* m+z− (*R_r_* = 0.33) adult hermaphrodite intestinal nuclei stained with α-DPY-27 (green), X-paint FISH probe (red) and DAPI (grayscale). (C) Summary of quantification of DPY-27 colocalization with the X chromosomes observed in wild type (mean *R_r_* = 0.70, n = 22), *htz-1(tm2469)*m+z− (mean *R_r_* = 0.45, n = 22) and *ssl-1(n4077)* m+z− (mean *R_r_* = 0.54, n = 21) adult hermaphrodite intestinal nuclei.

We hypothesized that the diffuse appearance of the DCC reflected mislocalization of the DCC away from the X chromosome. To observe DCC localization relative to X chromosomes, we combined DCC immunofluorescence with X-paint FISH in *htz-1(RNAi)* (Figure 4) and mutant (Figure 5) animals. We used intestinal nuclei because they are 32-ploid, allowing for easier visualization of sub-nuclear regions by FISH [92]. To determine the degree of colocalization between X-Paint and DPY-27 signals we determined Pearson's correlation coefficient (*R_r_*) values (see Materials and Methods). An *R_r_* value of +1 indicates a complete and positive correlation between two signals within a region of interest while a value of 0 indicates no linear relationship between the two signals.

In vector control RNAi animals we observed that the DCC was highly restricted to the X chromosomes and the mean *R_r_* was 0.65±0.14. *R_r_* was greater than 0.5 in the vast majority of nuclei observed (88%), and only a minority of nuclei had *R_r_* values between 0.5 and 0.2 (∼12%). No correlation values of less than 0.2 were observed in these animals. Representative nuclei and corresponding *R_r_* are shown in Figure 4A. By contrast, after *htz-1* RNAi, the mean *R_r_* for *htz-1* RNAi nuclei was 0.44±0.20, significantly lower than control (p = 5.79E-8). The majority of nuclei (58%) had DPY-27/X-paint correlation values below 0.5, and 27% of nuclei had values below the lowest value observed in the control. Representative *htz-1*(RNAi) nuclei and corresponding *R_r_* values are shown in Figure 4B. A summary of DPY-27/X-Paint colocalization quantification after vector and *htz-1* RNAi is shown in Figure 4C.

DCC mislocalization was also observed in intestinal nuclei of homozygous *htz-1(tm2469)* hermaphrodite progeny of heterozygous mothers (m+z−) (Figure 5) and in HTZ-1-depleted embryos (Figure S4). The mislocalization phenotype observed in *htz-1(tm2469)* (Figure 5) mutant animals was very similar to the observations made after *htz-1* RNAi. In wild-type hermaphrodites only 5% of nuclei observed had *R_r_* values below 0.5, but a majority of *htz-1(tm2469)* nuclei (59%) had values below 0.5. Additionally, 45% of nuclei observed had values below the lowest value observed in wild-type nuclei (Figure 5B and 5C). We also analyzed HTZ-1-depleted embryos after the 50-cell stage in development (after the onset of dosage compensation). We observed 16% of *htz-1*-depleted embryos with a diffuse nuclear DCC localization pattern (as opposed to 2% of vector RNAi control embryos), confirming that DCC mislocalization is not tissue specific (Figure S4).

Finally, we analyzed DCC distribution in nuclei of *ssl-1 (n4077)* mutant animals. *ssl-1* encodes a homolog of Swr1, the catalytic subunit of Swr1-com, the complex responsible for exchanging H2A for H2A.Z [93]–[96]. Consistent with this function, *ssl-1(n4077)* m+z− homozygous animals have reduced HTZ-1 staining (Figure 5A). In *ssl-1(n4077)* hermaphrodites, 43% of nuclei observed had *R_r_* values below 0.5, similar to what we observe after *htz-1* RNAi and in *htz-1(tm2469)* animals. Also, 33% of *ssl-1* nuclei had *R_r_* values below the lowest value observed in wild type nuclei, confirming that reduced HTZ-1 disrupts the localization of DCC to the X chromosomes (Figure 5B and 5C). Together, these results strongly suggest that HTZ-1, a protein more abundant on autosomes, is important for restricting localization of the DCC to the X chromosomes.

### DCC localization to autosomes upon HTZ-1 depletion

**Figure 6:**
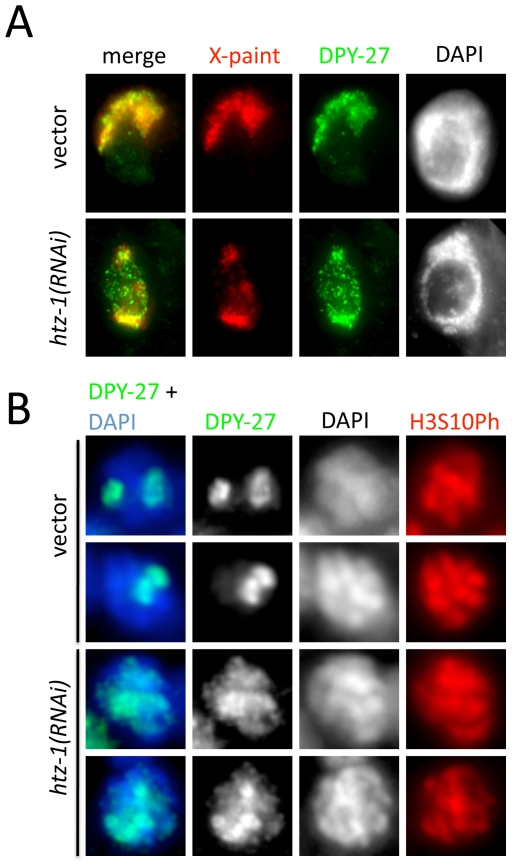
DCC associates with autosomes in HTZ-1-depleted cells. (A) DPY-27 (green) and X-chromosome (red) localization in vector and *htz-1* RNAi treated hermaphrodite adult intestinal nuclei after detergent extraction of nucleoplasmic proteins. DCC staining not in association with the X chromosomes remains after detergent extraction in *htz-1* RNAi animals. (B) DPY-27 (green) localization to prophase chromosomes (DAPI, blue) in vector and *htz-1* RNAi treated embryonic nuclei (>50 cell stage). DPY-27 staining to more than two chromosomes is observed in 32% of *htz-1(RNAi)* prophase nuclei (n = 63) but only observed in 11% of vector prophase nuclei (n = 57). Mitotic nuclei were identified using an antibody that recognizes H3S10Ph (red).

The portion of DCC which is not associated with the X chromosome appears nonetheless bound to chromatin. When we combined X-Paint FISH/DPY-27 IF with a protocol previously shown to extract nucleoplasmic proteins [97], we were unable to remove the non-X associated DCC within intestinal nuclei of *htz-1* RNAi animals (Figure 6A). This suggests that the non-X associated DCC is associated with autosomal chromatin.

To confirm DCC association with autosomes, we analyzed prophase chromosomes in both vector control and *htz-1* depleted embryos. We reasoned that individualized mitotic chromosomes would allow for more conspicuous visualization of DCC localization. To mark mitotic nuclei, embryos were co-stained with α-Phospho-Histone H3 Serine 10. In control embryos, DCC localization was largely restricted to two chromosomes in prophase nuclei. After *htz-1* RNAi, however, 32% of prophase nuclei had low-level DCC staining on more than two chromosomes (Figure 6B). These data indicate that the DCC associates not only with the X chromosome, but also with autosomes in HTZ-1-depleted animals. Taken together, these results suggest the existence of an HTZ-1 dependent autosomal repellent activity. In wild type animals, this activity restricts localization of the DCC to the X chromosome. Loss of *htz-1* reduces the efficiency of this repellant, allowing the DCC to bind other chromosomes.

### HTZ-1 levels are also reduced on the X chromosomes in the male and hermaphrodite germ lines

**Figure 7:**
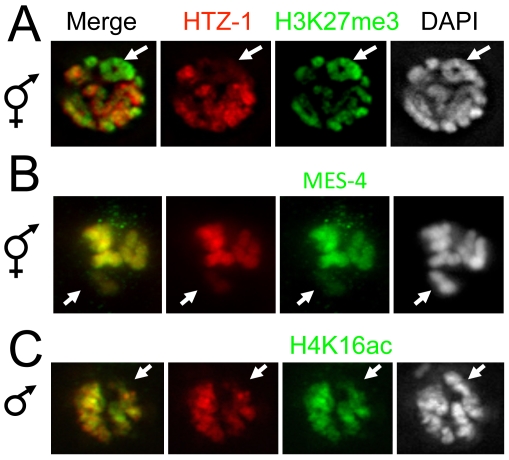
HTZ-1 is underrepresented on the silent X chromosomes of male and hermaphrodite germ lines. (A) Hermaphrodite germ nucleus in pachytene of prophase I of meiosis stained with antibodies specific to H3K27me3 (a mark enriched on the X, green), HTZ-1 (red), and DAPI (grayscale). (B) Hermaphrodite germ nucleus in diplotene of prophase I co-stained for MES-4 (enriched on autosomes, green), HTZ-1 (red), and DAPI (grayscale). (C) Male germ nucleus in pachytene of prophase I co-stained for H4K16ac (enriched on autosomes, green), HTZ-1 (red) and DAPI (grayscale). In all cases, HTZ-1 levels are lower on the X chromosome (arrows) than on autosomes.

In different organisms, H2A.Z has been observed to be either enriched in silent chromatin, (such as mammalian and *Drosophila* centromeres [77]–[80], or the XY sex body in the mammalian germ line [81]) or depleted in silent chromatin (such as heterochromatic chromocenters in plants [69],[83], or the transcriptionally inactive micronucleus in *Tetrahymena* [60]–[62]). Dosage compensation in worms is thought to involve two-fold downregulation of gene expression, but not complete silencing [2]. To explore whether HTZ-1 localizes to silent chromatin in worms, we examined its distribution in the germ line, where the X chromosomes are subject to chromosome-wide silencing by a mechanism unrelated to dosage compensation [50]. In the male germ line, the single X chromosome is subject to meiotic silencing of unpaired chromatin and is silent throughout meiosis. In the hermaphrodite germ line, the paired X chromosomes are silent during early meiosis, but become transcriptionally active in later stages [50]. To test HTZ-1 levels on the silent X chromosome in the germ line, we performed immunofluorescence experiments on dissected male and hermaphrodite gonads. To distinguish the X from autosomes we used antibodies specific to MES-4, H3K27me3, or H4K16ac, all of which have been used in previous studies to distinguish the X from autosomes in the germ line. MES-4, a SET-Domain protein, is enriched on autosomes and markedly depleted from the X chromosome in the germ line [51],[57]. Conversely, H3K27me3 is enriched on the silent X chromosomes in the germ line [53]. In the male germ line, H4K16ac is present on autosomes but absent from the unpaired X chromosome [50]. We found that HTZ-1 levels are much lower on the X chromosomes than on autosomes in both male and hermaphrodite germ lines (Figure 7). Thus, underrepresentation of HTZ-1 appears to be a general feature of both types of chromosome-wide repression in the worm: two-fold downregulation by dosage compensation and complete meiotic silencing. The possible involvement of HTZ-1 in germ line X chromosome silencing will be explored elsewhere.

## Discussion

Dosage compensation in *C. elegans* is accomplished by the DCC, a complex of proteins that binds the two X chromosomes in hermaphrodites to down-regulate expression of genes two-fold. In this study we report on the role of the histone variant H2A.Z/HTZ-1 in this process. HTZ-1 is less abundant on the dosage compensated X chromosomes in hermaphrodites but is found at higher levels on autosomes and X chromosomes in male somatic cells. When *htz-1* expression is reduced, levels of DCC proteins do not change. However, binding of the DCC is no longer restricted to the X chromosomes and dosage compensation is impaired.

### Models for HTZ-1 function in dosage compensation

**Figure 8:**
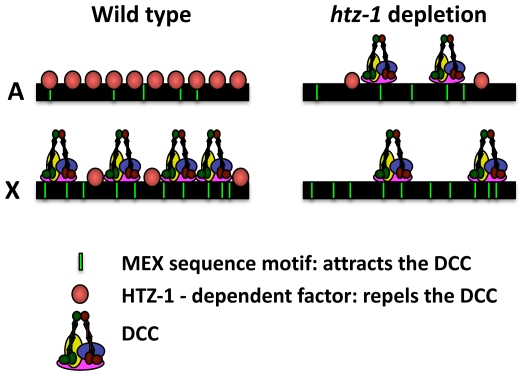
Model for HTZ-1 function in dosage compensation. DNA sequence motifs, which are enriched on the X chromosome (X), attract the DCC. HTZ-1, or a factor/activity dependent on HTZ-1, is enriched on autosomes (A) and repels the DCC. As a consequence of these two forces, in wild type cells the DCC is greatly enriched on the X chromosomes. However, when HTZ-1 levels are low, the DCC is now able to bind both the X chromosomes and the autosomes. Ectopic DCC binding on autosomes titrates the complex away from the X chromosomes and impairs dosage compensation.

One of the intriguing challenges in the study of dosage compensation is to understand how the DCC machinery is able to specifically target the X chromosomes for regulation. Our studies indicate that when HTZ-1 is depleted, the DCC appears to be no longer targeted correctly to the X chromosome. Rather than binding solely to the X chromosomes, the complex now binds autosomes as well. These results reveal that the normal function of HTZ-1 (or an HTZ-1 regulated factor) includes keeping the DCC away from autosomes. Previous studies indicated that specific DCC binding sites on the X chromosome, so-called *rex* sites (*r*ecruiting *e*lement on *X*), are important for attracting the DCC to the X chromosome ([44]–[47]). Taken together, these data suggest that positive forces (X-specific recruitment elements that attract the DCC) and negative forces (autosomal chromatin that repels the DCC) cooperate to discriminate the X from autosomes (Figure 8).

The mechanism of how HTZ-1 restricts DCC localization is unclear. We will consider three possible models. First, HTZ-1 may serve as a direct regulator of DCC binding. Targeting of the DCC to the X chromosome is believed to be a two-step process. The complex initially binds to an estimated 200 *rex* sites, followed by dispersal to numerous so-called *dox* sites (*d*ependent *o*n *X*) or “way stations” [44],[46],[47],[98]. *Rex* sites coincide with the highest peaks of DCC binding and are characterized by the presence and clustering of short sequence motifs called MEX motifs [46],[47],[99]. MEX motifs are slightly enriched on the X chromosome, but are also present on autosomes [46]. In principle, HTZ-1 can affect either DCC targeting to *rex* sites, or dispersal to *dox* sites, or both.

It should be pointed out that this model is different from the interpretation of the HTZ-1 localization data presented in [67]. Using high-resolution analysis of HTZ-1 binding, the authors showed that a subset of DCC peaks on the X chromosome coincide with HTZ-1 peaks. One way to reconcile their data and ours is to point out that the DCC/HTZ-1 overlap tends to be at promoters (DCC dox sites), and less so at the highest peaks of DCC binding (DCC foci or *rex* sites) [67]. Therefore, if HTZ-1 is a negative regulator of DCC binding, it is more likely that HTZ-1 affects the targeting step to *rex* sites, but not the dispersal step to *dox* sites. Another way to reconcile the data in the two studies is to suggest that HTZ-1 at *dox* sites is modified posttranslationally (see below) in such a way that permits DCC binding. According to this model, MEX motifs attract DCC to the X chromosome, whereas HTZ-1 negatively regulates DCC recruitment to *rex* sites. If a MEX motif-containing sequence is not bound by HTZ-1 the DCC will be recruited. However, if a MEX motif-containing sequence is bound by HTZ-1, the DCC will be prevented from binding. From sites of entry, the DCC then may be dispersed to *dox* sites in a sequence and HTZ-1 independent manner. When HTZ-1 levels are reduced by RNAi or mutation, the DCC will be able to bind all MEX motif containing sites, whether they are on the autosomes or on the X chromosome. Ectopic DCC binding to autosomes will reduce the amount of DCC binding to the X chromosomes, and dosage compensation will be impaired as a result. To test this model, it will be important to observe DCC binding patterns genome-wide at high resolution upon *htz-1* depletion and to determine whether ectopic DCC binding sites contain a DNA sequence motif similar to MEX motifs.

An alternative possibility is that changes in the higher order chromatin organization imposed by HTZ-1 determine whether the DCC is able to bind the chromosome. H2A.Z has been reported to alter the nucleosome surface, affect recruitment of other chromatin components, and thereby modulate higher order features of the chromatin fiber [77]. High levels of HTZ-1 on the autosomes may result in alterations in the overall structure of the chromatin fiber, which preclude DCC binding. Low levels of HTZ-1 on the X chromosome would allow DCC binding. Upon reduction of HTZ-1 levels, general disruption of higher order chromatin folding would allow the DCC to bind both the X and the autosomes. According to this model, the changes in chromatin fiber folding are a direct consequence of HTZ-1 levels on the chromosome. However, the model does not require complete mutually exclusive binding of the DCC and HTZ-1 at high resolution, and therefore does not conflict with the data in [67].

Finally, HTZ-1 may regulate expression of a DCC component, or another gene needed for proper DCC localization. While HTZ-1 is an obvious candidate for the DCC-repelling activity, it should be noted that in principle another HTZ-1-regulated protein could also perform this function. Our evidence, as yet, does not support this model, as we do not observe a change in DCC protein levels or *sdc-2* RNA levels when *htz-1* expression is reduced. In addition, most DCC proteins are loaded into oocytes, and this maternal load of DCC proteins is sufficient for healthy development. Therefore, it is unlikely that the observed dosage compensation defects in m+z− *htz-1* mutant animals are due to defects in transcription of DCC genes. However, it remains possible that HTZ-1 plays more subtle roles in regulating the exact levels and timing of expression of dosage compensation genes. Nonetheless, the difference in HTZ-1 levels in male and hermaphrodite X chromosomes (Figure 2C) argue for a more direct role for HTZ-1 in the hermaphrodite specific-process of dosage compensation. High-resolution analysis of HTZ-1 binding to the male X chromosome may help distinguish between the models presented above.

### HTZ-1 depletion on the X chromosome

A question that remains unanswered is how HTZ-1 is specifically targeted to autosomes, or conversely, how the X chromosomes become depleted of HTZ-1. The small number of developmentally important genes on the X chromosome relative to autosomes can certainly contribute to this difference [67]. However, this model does not explain why the male X in adult animals does not appear to be depleted of HTZ-1 at the chromosomal level (Figure 2C). The X chromosome in the both the male and hermaphrodite germ lines is subject to silencing [50]–[52], and it is possible that the chromosome maintains some memory of this silencing after fertilization. Such effects have been seen on the sperm-derived X chromosome [100]. The differences between the sperm derived X (which only hermaphrodite embryos receive) and the oocyte-derived X (which both males and hermaphrodites receive) may contribute to the sex-specific differences in observed X-linked HTZ-1 levels. Comparison of HTZ-1 dynamics in male and hermaphrodite embryos in early development will be an important future area of investigation.

### Different HTZ-1 pools for different functions

It is important to keep in mind that dosage compensation is a chromosome-wide gene regulation mechanism that is super-imposed on the unique transcriptional programs of individual X-linked genes. While HTZ-1 levels on the dosage compensated X chromosomes are reduced overall, the protein is not completely absent. Indeed, HTZ-1 binds to the promoter of an X-linked dosage compensated gene, *myo-2*, and is needed for its proper temporal activation [73]. It is possible that a pool of HTZ-1 functions at promoters, including promoters on the X chromosome, to promote timely regulation of gene expression. Superimposed on that is the global repression of the X chromosome by the DCC. Thus, HTZ-1 may perform a double role: it regulates genes both individually (by binding to promoters) and chromosome-wide (by regulating DCC binding).

Different pools of HTZ-1 may differ in their histone partners and/or the level of posttranslational modifications. Unlike yeast (where the only histone H3 is most similar to H3.3), worms possess both H3 and H3.3 [90]. Therefore, in principle, one population of HTZ-1 in worms is able to form labile nucleosomes, while another population can form stable nucleosomes. Furthermore, both populations can be modified by various posttranslational modifications, increasing the number of potentially different ways in which HTZ-1 can affect genome activity. Consistent with this idea, acetylation of the N-terminal tail of Htz1 is necessary for the anti-silencing property of Htz1 in *S. cerevisiae*, as unacetylatable Htz1 shows no change in localization to anti-silenced genes, but Sir complex spreading and decreased expression of anti-silenced genes is observed [101]. Htz1 is also subject to C-terminal SUMOylation. SUMO-Htz1 is implicated in directing chromosomes with persistent double-strand breaks to re-localize to the nuclear periphery in budding yeast [102]. Posttranslational modification of H2A.Z has also been observed in mammalian dosage compensation [25]. H2A.Z is under-represented on the inactive X in female mouse nuclei, but the small population of remaining H2A.Z is specifically mono-ubiquitylated by the Ring1b E3 ligase as part of the Polycomb repressor complex 1 (PRC1). Ring1b is also responsible for mono-ubiquitylation of histone H2A in X inactivation [24]. Although it is not currently understood how mono-ubiquitylation of H2A and H2A.Z function in X inactivation, the fact that this modification is largely specific to the inactive X suggests an important role. It is highly likely that *C. elegans* HTZ-1 is subject to posttranslational modification and it will be important to address how these modifications affect its role both in dosage compensation and in other processes.

### Barriers to repressor complex binding

The proposed role of HTZ-1 in dosage compensation is similar to that of two proteins shown to function in germ line X-chromosome silencing in *C. elegans*. The gene encoding MES-4 was originally identified in a forward genetic screen with several other genes whose mutations led to the same *mes* phenotype (*m*aternal *e*ffect *s*terility) [51]. MES-2, MES-3 and MES-6, are the protein products of the other genes identified, and these form a Polycomb repressor-like complex that is responsible for enriching the X chromosomes with the silencing H3K27me3 mark [53]. Surprisingly, MES-4, a histone H3 lysine 36 methyltransferase (HMT), localizes to autosomes, not the X, and yet it has been shown to be important for germ line X-chromosome silencing [51],[57]. MRG-1, an ortholog of the mammalian mortality factor related protein MRG15, is the second autosome-enriched protein that has been shown to play a role in germ line X chromosome silencing [103]. In both *mes-4* and *mrg-1* mutants, de-silencing of X-linked genes is observed. It has been proposed that the activities of MES-4 and MRG-1 on autosomes prevent the binding of a repressor protein or complex and help limit repressor binding to the X chromosomes. The proposed mode of action of MRG-1 and MES-4 in germ line X chromosome silencing is similar to the model of HTZ-1 function in dosage compensation we have proposed.

Our model describing HTZ-1 as an autosomal DCC barrier is also similar to the role of Htz1 in yeast in blocking the spread of silencing complexes into euchromatic regions adjacent to telomeres [74]. In *htz1Δ* cells, the Sir proteins spread into these regions, leading to silencing of genes. Recent evidence has shown that loss of Htz1 leads to ectopic Sir complex localization that is not limited to immediate anti-silenced regions, but, rather, is found throughout the genome [75]. Thus, Htz1p in yeast may serve a global, not just a local, anti-silencing function, similar to our proposed model of HTZ-1 action in worms. Furthermore, in *Arabidopsis*, H2A.Z also plays a global antisilencing role by protecting DNA from methylation [69]. When H2A.Z incorporation is compromised, DNA methylation expands into regions once protected by H2A.Z-containing nucleosomes. Therefore, a function for H2A.Z in the protection against transcriptional repression may be a widely conserved role for this histone variant.

Htz1 functions in parallel with other nucleosomal elements to prevent heterochromatic spreading. The Set1 complex is responsible for histone H3 lysine 4 methylation (H3K4me) and also has an anti-silencing function [104]. A recent study found that Set1 and Htz1 cooperate to mediate global antisilencing in yeast [75]. This raises the possibility that there are other nucleosomal modifications or elements that might function in X-chromosome DCC restriction in parallel with HTZ-1 in *C. elegans*.

## Materials and Methods

### Strains and alleles

All strains used were maintained as described [105]. Strains include: N2 Bristol strain (wild type), TY2384 *sex-1(y263)* X; TY4403 *him-8(e1489)* IV; *xol-1(y9) sex-1(y263)* X; EKM11 *htz-1(tm2469)* IV/*nT1(qIs51)* IV,V; MT12963 *ssl-1(n4077)III*/*eT1* (III;V); SM1353 *cha-1(p1182)* IV; *pxEx214(HTZ-1promoter::YFP::HTZ-1 + HTZ-1promoter::CFP::LacI +pRF4)* [73].

### RNA interference

*E. coli* HT115 bacteria expressing double stranded RNA for *htz-1*, *dpy-27*, *capg-1*, *his-71* (coding region), *his-24*, *hil-3*, *hil-4*, *hil-5*, *hil-6*, *hil-7*, *isw-1* or vector (polylinker), were used for feeding RNAi using the Ahringer feeding RNAi clones [106]. To generate RNAi vectors for *let-418* and the 3′ UTR of *his-71* and *his-72*, the regions were PCR amplified, digested with Bam HI and Bgl II (*let-418*) or Bgl II and Not I (*his-71* and *his-72*), and cloned into the DT7 vector as described [106]. The following primers were used for amplification:

*his-71* 3′-UTR

cgaagatctcgtgcataaacgttgagctg and gagcggccgccatgcacgctgttcaaaaac

*his-72* 3′-UTR

cgaagatctagctccatcaccaattctcg and gagcggccggcgtggaatatagttgct

*let-418*

catgggatccttgccgctcctcattcaact and gtacagatctgacgatgtgcacgagagaaa

RNAi in N2 was initiated at the L1–L2 stage. Adults were then transferred to new RNAi plates to produce progeny for 24 hours. For IF/FISH, western, and RT-PCR analysis, RNAi progeny were processed 24 hours post-L4. To score embryonic lethality in the *sex-1* strain, adult animals were allowed to lay eggs for 24 hours and the number of embryos laid was counted. The next day the number of dead embryos and larvae were counted and the percentage of embryonic lethality was calculated by dividing number of dead embryos by the total number of embryos laid. To score male rescue in *him-8(e1489) IV*; *xol-1(y9) sex-1(y263) X*, adult animals were allowed to lay eggs for 24 hours. When adult animals were removed from the RNAi plates, the number of embryos laid was counted. Three days later the number of male progeny on each plate was counted. Male viability was calculated by dividing the number of male progeny observed by the expected number of males. The *him-8(e1489)* mutation reproducibly results in 38% male self-progeny [89], so the expected number of males was determined to be 38% of total embryos laid. Male rescue = Number of males/(total embryos×0.38). All RNAi was conducted at 20°C.

### Immunostaining

Rabbit and rat α-HTZ-1 antibodies were raised against the C-terminal 19 amino acids (NKKGAPVPGKPGAPGQGPQ) and affinity purified. Polyclonal rat α-HTZ-1 was used at a dilution of 1∶500, polyclonal rabbit α-HTZ-1 at a dilution of 1∶100 for immunofluorescence. Other primary antibodies used are: polyclonal rabbit α-DPY-27 at a dilution of 1∶100 [38], polyclonal rabbit α-MES-4 (Susan Strome [UC Santa Cruz], [57]) at 1∶100, rabbit antiserum α-acetyl-histone H4 (Lys16) (Upstate) at 1∶100, rabbit polyclonal α-trimethyl-histone H3 (Lys27) (Upstate) at 1∶500, and mouse monoclonal α-phospho-histone H3S10 (6G3) (Cell Signaling Technology) at 1∶500. Secondary antibodies used are: Fluorescein (FITC) conjugated donkey α-rabbit (Jackson ImmunoResearch) and Cy3 conjugated donkey α-rabbit IgG (Jackson ImmunoResearch) both at a dilution of 1∶100. Embryos were stained as described [34]. Adult animals were dissected and stained as described [44]. In adults, somatic non-intestinal nuclei near the cut site (vulval area) were observed. Images were captured with a Hamamatsu ORCA-ERGA CCD camera mounted on an Olympus BX61 motorized X-drive microscope using a 60× oil immersion objective. Captured images were deconvolved using 3i Slidebook imaging software. Projected images were taken at 0.2 µm intervals through samples. Adobe Photoshop was used for assembling images.

### Fluorescent *in situ* hybridization

FISH probe templates were generated by degenerate oligonucleotide primed PCR to amplify purified yeast artificial chromosome DNA. The labeled X-paint probe was prepared and used as described [44]. Hybridization was performed on adult animals (24 hours post-L4) with or without previous RNAi treatment. For X-paint hybridization followed by DPY-27 immunostaining, sample and probe were denatured at 95°C for 3 minutes. For X-paint hybridization followed by HTZ-1 immunostaining, sample and probe were denatured at 78–80°C for 8–10 minutes in a Hybaid OmniSlide *in situ* Thermal Cycler System (Thermo Scientific). Imaging was conducted as described above.

### Quantification of colocalization

3i Slidebook imaging software was used to measure colocalization of DPY-27 (FITC) and X-Paint (Cy3) signals on images obtained as described above. A FITC mask was set for each nucleus z-stack and the correlation between signals was calculated within this mask by the software. The FITC∶Cy3 correlation coefficient was recorded and used as an indication of colocalization between DPY-27 and X-Paint.

### Detergent extraction

Detergent extraction of nucleoplasmic protein from dissected nuclei was performed by dissecting animals in 1× sperm salts plus 1% Triton detergent [97]. Dissected animals were then processed for either Fluorescent *in situ* hybridization or immunofluorescence.

### Western blot analysis

For each treatment described, 100 animals (all 24 hours post-L4) were picked into 1XM9, washed, and incubated for ten minutes at 95°C in 19 µl SDS-PAGE loading dye (0.1 M Tris-HCl pH 6.8, 75 M Urea, 2% SDS, Bromophenol Blue for color) plus 1 µl β-mercaptoethanol. The treated samples were then loaded into either 6% acrylamide (for detection of DPY-27, MIX-1, DPY-26, DPY-28, and CAPG-1) or 15% acrylamide gels (for detection of HTZ-1). SDS-PAGE was performed and protein was transferred onto nitrocellulose. The following antibodies and dilutions were used: rabbit α-HTZ-1 at 1∶500, rabbit α-DPY-27 at 1∶500, rabbit α-CAPG-1 at 1∶500 [38], rabbit α-DPY-28 (gift of K. Hagstrom) at 1∶500, rabbit α-DPY-26 (gift of K. Hagstrom) at 1∶5000, rabbit α-MIX-1 (gift of R. Chan) at 1∶500, mouse monoclonal α- α-Tubulin (Sigma) at 1∶1000, LI-COR IRDye 800CW Conjugated Goat (polyclonal) α-Mouse IgG at 1∶10000, LI-COR IRDye 800CW Conjugated Goat (polyclonal) α-Rabbit IgG at 1∶10000. Blots were scanned and band intensities were quantified using an Odyssey Infrared Imaging System (LI-COR Biosciences). Protein levels for DCC proteins and HTZ-1 were normalized to α-tubulin. Relative protein levels after *htz-1* RNAi were calculated by dividing the normalized *htz-1* RNAi level by normalized vector RNAi level.

### RT–PCR

Trizol (Invitrogen) was used to extract RNA from all samples. Worms were washed from RNAi plates or normal OP50 plates 24 hours post L4, washed with M9 and stored at −80°C until extraction. For RNA extraction, samples were thawed on ice and tissue was homogenized by grinding using a microcentrifuge tube pestle. Tissue was ground in three 60-second intervals and re-frozen in liquid nitrogen between each interval. During the final 60-second interval, 250 µl of Trizol was added to the tube, and when completed, another 250 µl was added for a total volume of 500 µl Trizol and the standard protocol was used to extract RNA from the homogenized samples (Invitrogen). DNA-Free kit (Applied Biosystems) was used to digest remaining DNA contamination.

Reverse transcription (RT) reactions were performed utilizing the High Capacity cDNA Reverse Transcription Kit with RNase Inhibitor (Applied Biosystems). 1 µl of DNase-treated RNA was used in each RT reaction.

PCR was used to observe relative levels of *htz-1*, R08C7.10, and *act-1* (actin) expression levels. The following primers were used with a 60°C annealing temperature:

*act-1*: gctatgttccagccatccttc and aagagcggtgatttccttctg

*htz-1*: tggctggaggaaaaggaaag and aacgatggatgtgtgggatg

R08C7.10: gtagaccaaaccagccagca and agcgccttgacgatacttttt

### Real-time PCR analysis

Real-time PCR analysis was conducted as described [98]. The following primers were used with an annealing temperature of 59°C:

*act-1*: same as above

*htz-1*: gcgctgccatcctcgaat and gggctcccttcttgttcatc

*sdc-2*: ggaaacaagaccgacaggaa and gatgcaatagtacacgccaaatc

Relative *htz-1* and *sdc-1* expression levels were calculated using the Pfaffl method [107] incorporating the PCR efficiency for each primer set as determined by a 10-fold dilution series for each primer set in each reaction. Reactions were conducted in triplicate per experiment. Data shown are resulting averages from three experiments.

## Supporting Information

Figure S1 α-HTZ-1 antibody is specific. (A) HTZ-1 (red), DPY-27 (green), and DAPI (grayscale) staining of adult intestinal nuclei in vector and *htz-1* RNAi treated hermaphrodites. The HTZ-1 signal is greatly reduced after *htz-1* RNAi. (B) α-HTZ-1 Western blot. α-HTZ-1 recognizes a band of the expected size (∼15 kDa) in both embryonic extract and adult protein samples. The α-HTZ-1 signal is reduced in *htz-1* RNAi treated animals as compared to vector treated animals.
(7.19 MB TIF)

Figure S2 Transgenic YFP-HTZ-1 is also under-represented on the dosage compensated X chromosomes. Embryos after the onset of dosage compensation were stained with α-GFP antibodies to observe YFP-HTZ-1 localization (red), α-DPY-27 to mark the X chromosome (green) and DAPI (grayscale). Arrows in the top panels indicate enlarged nucleus shown below. YFP-HTZ-1 levels are reduced in the territory of the dosage compensated X chromosomes.
(7.78 MB TIF)

Figure S3 tm2469 deletion affects *htz-1* and R08C7.10 expression. (A) Reverse-transcription polymerase chain reaction (RT-PCR) analysis of expression of *htz-1*, R08C7.10 and actin (control) in wild type (+/+), heterozygous (*htz-1(tm2469)/nT1*), or homozygous (*htz-1(tm2469)*) animals. Expression of both *htz-1* and R08C7.10 is affected in homozygous animals. Contaminating amplification product from residual DNA in the RNA sample is indicated by a star. (B) Schematic showing relative positions of *htz-1* and R08C7.10 on chromosome IV (not to scale). The tm2469 deletion removes most of the coding region of *htz-1*. In addition, it likely the affects the promoter or other *cis* control elements of the R08C7.10, a gene located only 522 base pairs away from the deletion.
(5.72 MB TIF)

Figure S4 *htz-1*-depleted embryos also show compromised DCC localization. Vector and *htz-1* RNAi embryos were stained with α-Phospho-H3 Ser10 (red) (to mark mitotic nuclei) and α-DPY-27 (green). After htz-1 RNAi, 16% of embryos with Phospho-H3 Ser10 staining (>50 cell stage) had diffuse nuclear DPY-27 localization (n = 372), as opposed to 2% in vector embryos (n = 314).
(2.89 MB TIF)

Table S1 RNAi of the following genes did not result in significant (>10%) male rescue.
(0.12 MB DOC)

## References

[1] Mendjan S, Akhtar A (2007). The right dose for every sex.. Chromosoma.

[2] Meyer BJ, *The C. elegans* Research Community, editor (2005). X-Chromosome dosage compensation.. http://wormbook.org.

[3] Payer B, Lee JT (2008). X Chromosome Dosage Compensation: How Mammals Keep the Balance.. Annu Rev Genet.

[4] Straub T, Becker PB (2007). Dosage compensation: the beginning and end of generalization.. Nat Rev Genet.

[5] Lucchesi JC, Kelly WG, Panning B (2005). Chromatin Remodeling in Dosage Compensation.. Annu Rev Genet.

[6] Hamada FN, Park PJ, Gordadze PR, Kuroda MI (2005). Global regulation of X chromosomal genes by the MSL complex in Drosophila melanogaster.. Genes Dev.

[7] Straub T, Gilfillan GD, Maier VK, Becker PB (2005). The Drosophila MSL complex activates the transcription of target genes.. Genes Dev.

[8] Palmer MJ, Mergner VA, Richman R, Manning JE, Kuroda MI (1993). The male-specific lethal-one (msl-1) gene of Drosophila melanogaster encodes a novel protein that associates with the X chromosome in males.. Genetics.

[9] Zhou S, Yang Y, Scott MJ, Pannuti A, Fehr KC (1995). Male-specific lethal 2, a dosage compensation gene of Drosophila, undergoes sex-specific regulation and encodes a protein with a RING finger and a metallothionein-like cysteine cluster.. Embo J.

[10] Gorman M, Franke A, Baker BS (1995). Molecular characterization of the male-specific lethal-3 gene and investigations of the regulation of dosage compensation in Drosophila.. Development.

[11] Kuroda MI, Kernan MJ, Kreber R, Ganetzky B, Baker BS (1991). The maleless protein associates with the X chromosome to regulate dosage compensation in Drosophila.. Cell.

[12] Gu W, Szauter P, Lucchesi JC (1998). Targeting of MOF, a putative histone acetyl transferase, to the X chromosome of Drosophila melanogaster.. Dev Genet.

[13] Amrein H, Axel R (1997). Genes expressed in neurons of adult male Drosophila.. Cell.

[14] Meller VH, Wu KH, Roman G, Kuroda MI, Davis RL (1997). roX1 RNA paints the X chromosome of male Drosophila and is regulated by the dosage compensation system.. Cell.

[15] Turner BM, Birley AJ, Lavender J (1992). Histone H4 isoforms acetylated at specific lysine residues define individual chromosomes and chromatin domains in Drosophila polytene nuclei.. Cell.

[16] Akhtar A, Becker PB (2000). Activation of transcription through histone H4 acetylation by MOF, an acetyltransferase essential for dosage compensation in Drosophila.. Mol Cell.

[17] Hilfiker A, Hilfiker-Kleiner D, Pannuti A, Lucchesi JC (1997). mof, a putative acetyl transferase gene related to the Tip60 and MOZ human genes and to the SAS genes of yeast, is required for dosage compensation in Drosophila.. Embo J.

[18] Kind J, Vaquerizas JM, Gebhardt P, Gentzel M, Luscombe NM (2008). Genome-wide analysis reveals MOF as a key regulator of dosage compensation and gene expression in Drosophila.. Cell.

[19] Borsani G, Tonlorenzi R, Simmler MC, Dandolo L, Arnaud D (1991). Characterization of a murine gene expressed from the inactive X chromosome.. Nature.

[20] Brockdorff N, Ashworth A, Kay GF, Cooper P, Smith S (1991). Conservation of position and exclusive expression of mouse Xist from the inactive X chromosome.. Nature.

[21] Brown CJ, Ballabio A, Rupert JL, Lafreniere RG, Grompe M (1991). A gene from the region of the human X inactivation centre is expressed exclusively from the inactive X chromosome.. Nature.

[22] Clemson CM, McNeil JA, Willard HF, Lawrence JB (1996). XIST RNA paints the inactive X chromosome at interphase: evidence for a novel RNA involved in nuclear/chromosome structure.. J Cell Biol.

[23] Plath K, Fang J, Mlynarczyk-Evans SK, Cao R, Worringer KA (2003). Role of histone H3 lysine 27 methylation in X inactivation.. Science.

[24] de Napoles M, Mermoud JE, Wakao R, Tang YA, Endoh M (2004). Polycomb group proteins Ring1A/B link ubiquitylation of histone H2A to heritable gene silencing and X inactivation.. Dev Cell.

[25] Sarcinella E, Zuzarte PC, Lau PN, Draker R, Cheung P (2007). Monoubiquitylation of H2A.Z distinguishes its association with euchromatin or facultative heterochromatin.. Mol Cell Biol.

[26] Boggs BA, Cheung P, Heard E, Spector DL, Chinault AC (2002). Differentially methylated forms of histone H3 show unique association patterns with inactive human X chromosomes.. Nat Genet.

[27] Costanzi C, Pehrson JR (1998). Histone macroH2A1 is concentrated in the inactive X chromosome of female mammals.. Nature.

[28] Chadwick BP, Willard HF (2003). Chromatin of the Barr body: histone and non-histone proteins associated with or excluded from the inactive X chromosome.. Hum Mol Genet.

[29] Chaumeil J, Okamoto I, Guggiari M, Heard E (2002). Integrated kinetics of X chromosome inactivation in differentiating embryonic stem cells.. Cytogenet Genome Res.

[30] Belyaev N, Keohane AM, Turner BM (1996). Differential underacetylation of histones H2A, H3 and H4 on the inactive X chromosome in human female cells.. Hum Genet.

[31] Boggs BA, Connors B, Sobel RE, Chinault AC, Allis CD (1996). Reduced levels of histone H3 acetylation on the inactive X chromosome in human females.. Chromosoma.

[32] Jeppesen P, Turner BM (1993). The inactive X chromosome in female mammals is distinguished by a lack of histone H4 acetylation, a cytogenetic marker for gene expression.. Cell.

[33] Bernstein E, Muratore-Schroeder TL, Diaz RL, Chow JC, Changolkar LN (2008). A phosphorylated subpopulation of the histone variant macroH2A1 is excluded from the inactive X chromosome and enriched during mitosis.. Proc Natl Acad Sci U S A.

[34] Chuang PT, Albertson DG, Meyer BJ (1994). DPY-27:a chromosome condensation protein homolog that regulates C. elegans dosage compensation through association with the X chromosome.. Cell.

[35] Lieb JD, Albrecht MR, Chuang PT, Meyer BJ (1998). MIX-1: an essential component of the C. elegans mitotic machinery executes X chromosome dosage compensation.. Cell.

[36] Lieb JD, Capowski EE, Meneely P, Meyer BJ (1996). DPY-26, a link between dosage compensation and meiotic chromosome segregation in the nematode.. Science.

[37] Tsai CJ, Mets DG, Albrecht MR, Nix P, Chan A (2008). Meiotic crossover number and distribution are regulated by a dosage compensation protein that resembles a condensin subunit.. Genes Dev.

[38] Csankovszki G, Collette K, Spahl K, Carey J, Snyder M (2009). Three distinct condensin complexes control C. elegans chromosome dynamics.. Curr Biol.

[39] Dawes HE, Berlin DS, Lapidus DM, Nusbaum C, Davis TL (1999). Dosage compensation proteins targeted to X chromosomes by a determinant of hermaphrodite fate.. Science.

[40] Davis TL, Meyer BJ (1997). SDC-3 coordinates the assembly of a dosage compensation complex on the nematode X chromosome.. Development.

[41] Hsu DR, Chuang PT, Meyer BJ (1995). DPY-30, a nuclear protein essential early in embryogenesis for Caenorhabditis elegans dosage compensation.. Development.

[42] Chu DS, Dawes HE, Lieb JD, Chan RC, Kuo AF (2002). A molecular link between gene-specific and chromosome-wide transcriptional repression.. Genes Dev.

[43] Villeneuve AM, Meyer BJ (1987). sdc-1: a link between sex determination and dosage compensation in C. elegans.. Cell.

[44] Csankovszki G, McDonel P, Meyer BJ (2004). Recruitment and spreading of the C. elegans dosage compensation complex along X chromosomes.. Science.

[45] Ercan S, Giresi PG, Whittle CM, Zhang X, Green RD (2007). X chromosome repression by localization of the C. elegans dosage compensation machinery to sites of transcription initiation.. Nat Genet.

[46] Jans J, Gladden JM, Ralston EJ, Pickle CS, Michel AH (2009). A condensin-like dosage compensation complex acts at a distance to control expression throughout the genome.. Genes Dev.

[47] McDonel P, Jans J, Peterson BK, Meyer BJ (2006). Clustered DNA motifs mark X chromosomes for repression by a dosage compensation complex.. Nature.

[48] Meyer BJ, Casson LP (1986). Caenorhabditis elegans compensates for the difference in X chromosome dosage between the sexes by regulating transcript levels.. Cell.

[49] Hirano T (2005). Condensins: organizing and segregating the genome.. Curr Biol.

[50] Kelly WG, Schaner CE, Dernburg AF, Lee MH, Kim SK (2002). X-chromosome silencing in the germ line of C. elegans.. Development.

[51] Fong Y, Bender L, Wang W, Strome S (2002). Regulation of the different chromatin states of autosomes and X chromosomes in the germ line of C. elegans.. Science.

[52] Garvin C, Holdeman R, Strome S (1998). The phenotype of mes-2, mes-3, mes-4 and mes-6, maternal-effect genes required for survival of the germ line in Caenorhabditis elegans, is sensitive to chromosome dosage.. Genetics.

[53] Bender LB, Cao R, Zhang Y, Strome S (2004). The MES-2/MES-3/MES-6 complex and regulation of histone H3 methylation in C. elegans.. Curr Biol.

[54] Holdeman R, Nehrt S, Strome S (1998). MES-2, a maternal protein essential for viability of the germ line in Caenorhabditis elegans, is homologous to a Drosophila Polycomb group protein.. Development.

[55] Korf I, Fan Y, Strome S (1998). The Polycomb group in Caenorhabditis elegans and maternal control of germ line development.. Development.

[56] Xu L, Fong Y, Strome S (2001). The Caenorhabditis elegans maternal-effect sterile proteins, MES-2, MES-3, and MES-6, are associated in a complex in embryos.. Proc Natl Acad Sci U S A.

[57] Bender LB, Suh J, Carroll CR, Fong Y, Fingerman IM (2006). MES-4: an autosome-associated histone methyltransferase that participates in silencing the X chromosomes in the C. elegans germ line.. Development.

[58] Reuben M, Lin R (2002). Germ line X chromosomes exhibit contrasting patterns of histone H3 methylation in Caenorhabditis elegans.. Dev Biol.

[59] Jin C, Felsenfeld G (2007). Nucleosome stability mediated by histone variants H3.3 and H2A.Z.. Genes Dev.

[60] Allis CD, Richman R, Gorovsky MA, Ziegler YS, Touchstone B (1986). hv1 is an evolutionarily conserved H2A variant that is preferentially associated with active genes.. J Biol Chem.

[61] Stargell LA, Bowen J, Dadd CA, Dedon PC, Davis M (1993). Temporal and spatial association of histone H2A variant hv1 with transcriptionally competent chromatin during nuclear development in Tetrahymena thermophila.. Genes Dev.

[62] Wenkert D, Allis CD (1984). Timing of the appearance of macronuclear-specific histone variant hv1 and gene expression in developing new macronuclei of Tetrahymena thermophila.. J Cell Biol.

[63] Guillemette B, Bataille AR, Gevry N, Adam M, Blanchette M (2005). Variant histone H2A.Z is globally localized to the promoters of inactive yeast genes and regulates nucleosome positioning.. PLoS Biol.

[64] Li B, Pattenden SG, Lee D, Gutierrez J, Chen J (2005). Preferential occupancy of histone variant H2AZ at inactive promoters influences local histone modifications and chromatin remodeling.. Proc Natl Acad Sci U S A.

[65] Raisner RM, Hartley PD, Meneghini MD, Bao MZ, Liu CL (2005). Histone variant H2A.Z marks the 5′ ends of both active and inactive genes in euchromatin.. Cell.

[66] Zhang H, Roberts DN, Cairns BR (2005). Genome-wide dynamics of Htz1, a histone H2A variant that poises repressed/basal promoters for activation through histone loss.. Cell.

[67] Whittle CM, McClinic KN, Ercan S, Zhang X, Green RD (2008). The genomic distribution and function of histone variant HTZ-1 during C. elegans embryogenesis.. PLoS Genet.

[68] Mavrich TN, Jiang C, Ioshikhes IP, Li X, Venters BJ (2008). Nucleosome organization in the Drosophila genome.. Nature.

[69] Zilberman D, Coleman-Derr D, Ballinger T, Henikoff S (2008). Histone H2A.Z and DNA methylation are mutually antagonistic chromatin marks.. Nature.

[70] Barski A, Cuddapah S, Cui K, Roh TY, Schones DE (2007). High-resolution profiling of histone methylations in the human genome.. Cell.

[71] Adam M, Robert F, Larochelle M, Gaudreau L (2001). H2A.Z is required for global chromatin integrity and for recruitment of RNA polymerase II under specific conditions.. Mol Cell Biol.

[72] Brickner DG, Cajigas I, Fondufe-Mittendorf Y, Ahmed S, Lee PC (2007). H2A.Z-mediated localization of genes at the nuclear periphery confers epigenetic memory of previous transcriptional state.. PLoS Biol.

[73] Updike DL, Mango SE (2006). Temporal regulation of foregut development by HTZ-1/H2A.Z and PHA-4/FoxA.. PLoS Genet.

[74] Meneghini MD, Wu M, Madhani HD (2003). Conserved histone variant H2A.Z protects euchromatin from the ectopic spread of silent heterochromatin.. Cell.

[75] Venkatasubrahmanyam S, Hwang WW, Meneghini MD, Tong AH, Madhani HD (2007). Genome-wide, as opposed to local, antisilencing is mediated redundantly by the euchromatic factors Set1 and H2A.Z.. Proc Natl Acad Sci U S A.

[76] Bruce K, Myers FA, Mantouvalou E, Lefevre P, Greaves I (2005). The replacement histone H2A.Z in a hyperacetylated form is a feature of active genes in the chicken.. Nucleic Acids Res.

[77] Fan JY, Rangasamy D, Luger K, Tremethick DJ (2004). H2A.Z alters the nucleosome surface to promote HP1alpha-mediated chromatin fiber folding.. Mol Cell.

[78] Greaves IK, Rangasamy D, Ridgway P, Tremethick DJ (2007). H2A.Z contributes to the unique 3D structure of the centromere.. Proc Natl Acad Sci U S A.

[79] Rangasamy D, Berven L, Ridgway P, Tremethick DJ (2003). Pericentric heterochromatin becomes enriched with H2A.Z during early mammalian development.. Embo J.

[80] Swaminathan J, Baxter EM, Corces VG (2005). The role of histone H2Av variant replacement and histone H4 acetylation in the establishment of Drosophila heterochromatin.. Genes Dev.

[81] Greaves IK, Rangasamy D, Devoy M, Marshall Graves JA, Tremethick DJ (2006). The X and Y chromosomes assemble into H2A.Z-containing [corrected] facultative heterochromatin [corrected] following meiosis.. Mol Cell Biol.

[82] Chadwick BP, Willard HF (2001). Histone H2A variants and the inactive X chromosome: identification of a second macroH2A variant.. Hum Mol Genet.

[83] Deal RB, Topp CN, McKinney EC, Meagher RB (2007). Repression of flowering in Arabidopsis requires activation of FLOWERING LOCUS C expression by the histone variant H2A.Z.. Plant Cell.

[84] Cui M, Han M (2007). Roles of chromatin factors in C. elegans development.. WormBook:.

[85] Carmi I, Kopczynski JB, Meyer BJ (1998). The nuclear hormone receptor SEX-1 is an X-chromosome signal that determines nematode sex.. Nature.

[86] Carmi I, Meyer BJ (1999). The primary sex determination signal of Caenorhabditis elegans.. Genetics.

[87] Gladden JM, Farboud B, Meyer BJ (2007). Revisiting the X:A signal that specifies Caenorhabditis elegans sexual fate.. Genetics.

[88] Miller LM, Plenefisch JD, Casson LP, Meyer BJ (1988). xol-1: a gene that controls the male modes of both sex determination and X chromosome dosage compensation in C. elegans.. Cell.

[89] Hodgkin J, Horvitz HR, Brenner S (1979). Nondisjunction Mutants of the Nematode CAENORHABDITIS ELEGANS.. Genetics.

[90] Ooi SL, Priess JR, Henikoff S (2006). Histone H3.3 variant dynamics in the germ line of Caenorhabditis elegans.. PLoS Genet.

[91] Jedrusik MA, Schulze E (2001). A single histone H1 isoform (H1.1) is essential for chromatin silencing and germ line development in Caenorhabditis elegans.. Development.

[92] Hedgecock EM, White JG (1985). Polyploid tissues in the nematode Caenorhabditis elegans.. Dev Biol.

[93] Kobor MS, Venkatasubrahmanyam S, Meneghini MD, Gin JW, Jennings JL (2004). A protein complex containing the conserved Swi2/Snf2-related ATPase Swr1p deposits histone variant H2A.Z into euchromatin.. PLoS Biol.

[94] Krogan NJ, Baetz K, Keogh MC, Datta N, Sawa C (2004). Regulation of chromosome stability by the histone H2A variant Htz1, the Swr1 chromatin remodeling complex, and the histone acetyltransferase NuA4.. Proc Natl Acad Sci U S A.

[95] Krogan NJ, Keogh MC, Datta N, Sawa C, Ryan OW (2003). A Snf2 family ATPase complex required for recruitment of the histone H2A variant Htz1.. Mol Cell.

[96] Mizuguchi G, Shen X, Landry J, Wu WH, Sen S (2004). ATP-driven exchange of histone H2AZ variant catalyzed by SWR1 chromatin remodeling complex.. Science.

[97] Chan RC, Severson AF, Meyer BJ (2004). Condensin restructures chromosomes in preparation for meiotic divisions.. J Cell Biol.

[98] Blauwkamp TA, Csankovszki G (2009). Two classes of dosage compensation complex binding elements along Caenorhabditis elegans X chromosomes.. Mol Cell Biol.

[99] Ercan S, Lieb JD (2009). C. elegans dosage compensation: a window into mechanisms of domain-scale gene regulation.. Chromosome Res.

[100] Bean CJ, Schaner CE, Kelly WG (2004). Meiotic pairing and imprinted X chromatin assembly in Caenorhabditis elegans.. Nat Genet.

[101] Babiarz JE, Halley JE, Rine J (2006). Telomeric heterochromatin boundaries require NuA4-dependent acetylation of histone variant H2A.Z in Saccharomyces cerevisiae.. Genes Dev.

[102] Kalocsay M, Hiller NJ, Jentsch S (2009). Chromosome-wide Rad51 spreading and SUMO-H2A.Z-dependent chromosome fixation in response to a persistent DNA double-strand break.. Mol Cell.

[103] Takasaki T, Liu Z, Habara Y, Nishiwaki K, Nakayama J (2007). MRG-1, an autosome-associated protein, silences X-linked genes and protects germ line immortality in Caenorhabditis elegans.. Development.

[104] Krogan NJ, Dover J, Khorrami S, Greenblatt JF, Schneider J (2002). COMPASS, a histone H3 (Lysine 4) methyltransferase required for telomeric silencing of gene expression.. J Biol Chem.

[105] Brenner S (1974). The genetics of Caenorhabditis elegans.. Genetics.

[106] Ahringer JE, The *C. elegans* Research Community, editor. (2005). Reverse genetics.. http://www.wormbook.org.

[107] Pfaffl MW (2001). A new mathematical model for relative quantification in real-time RT-PCR.. Nucleic Acids Res.

