# Supplementary figures and images for: Restricting Dosage Compensation Complex Binding to the X Chromosomes by H2A.Z/HTZ-1
Source: PLoS Genet. 2009 Oct 23;5(10):e1000699. doi: 10.1371/journal.pgen.1000699 (PMC2760203; doi:10.1371/journal.pgen.1000699)

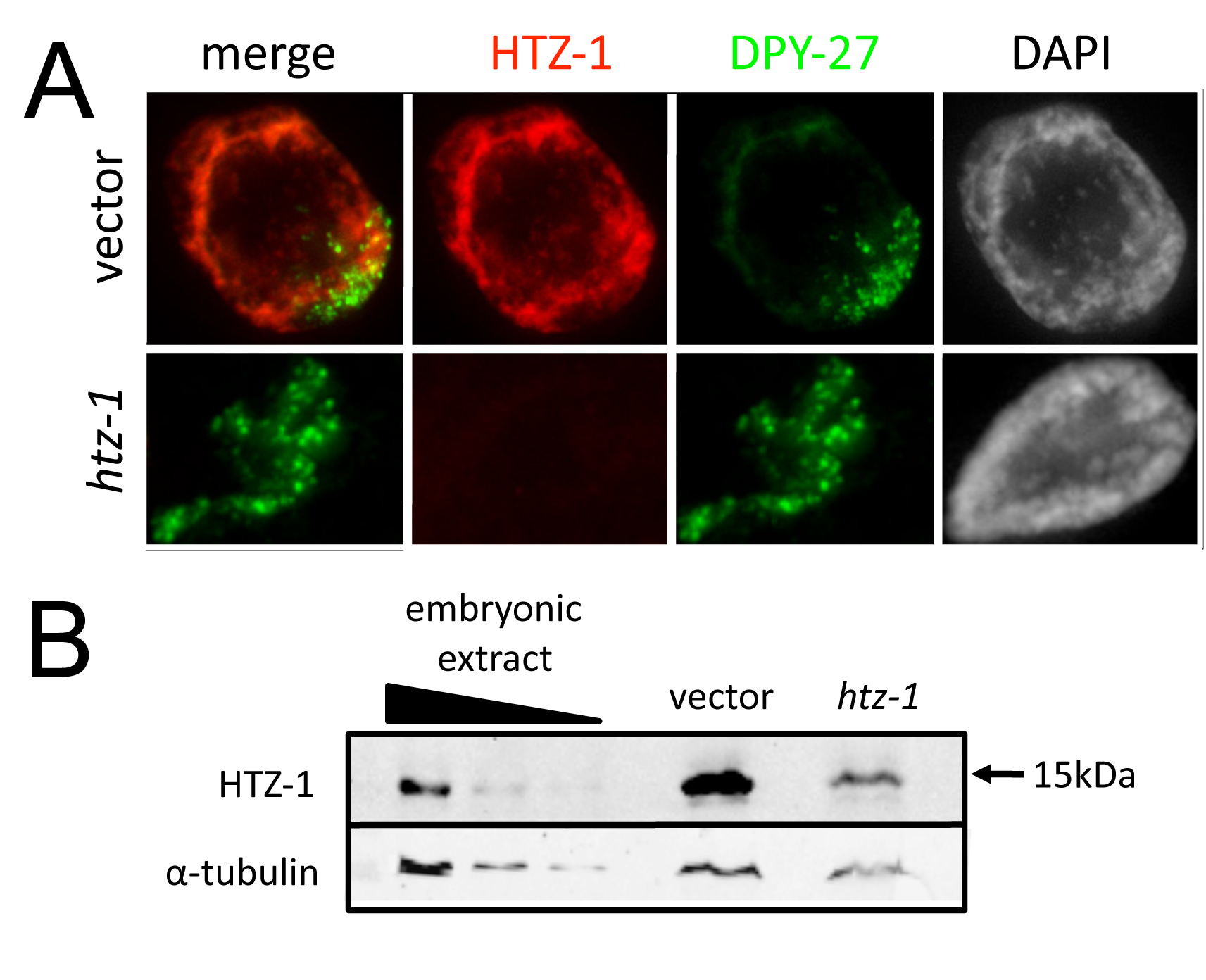

Supplement: Figure S1 — α-HTZ-1 antibody is specific. (A) HTZ-1 (red), DPY-27 (green), and DAPI (grayscale) staining of adult intestinal nuclei in vector and htz-1 RNAi treated hermaphrodites. The HTZ-1 signal is greatly reduced after htz-1 RNAi. (B) α-HTZ-1 Western blot. α-HTZ-1 recognizes a band of the expected size (∼15 kDa) in both embryonic extract and adult protein samples. The α-HTZ-1 signal is reduced in htz-1 RNAi treated animals as compared to vector treated animals. (7.19 MB TIF) [file pgen.1000699.s001.tif]

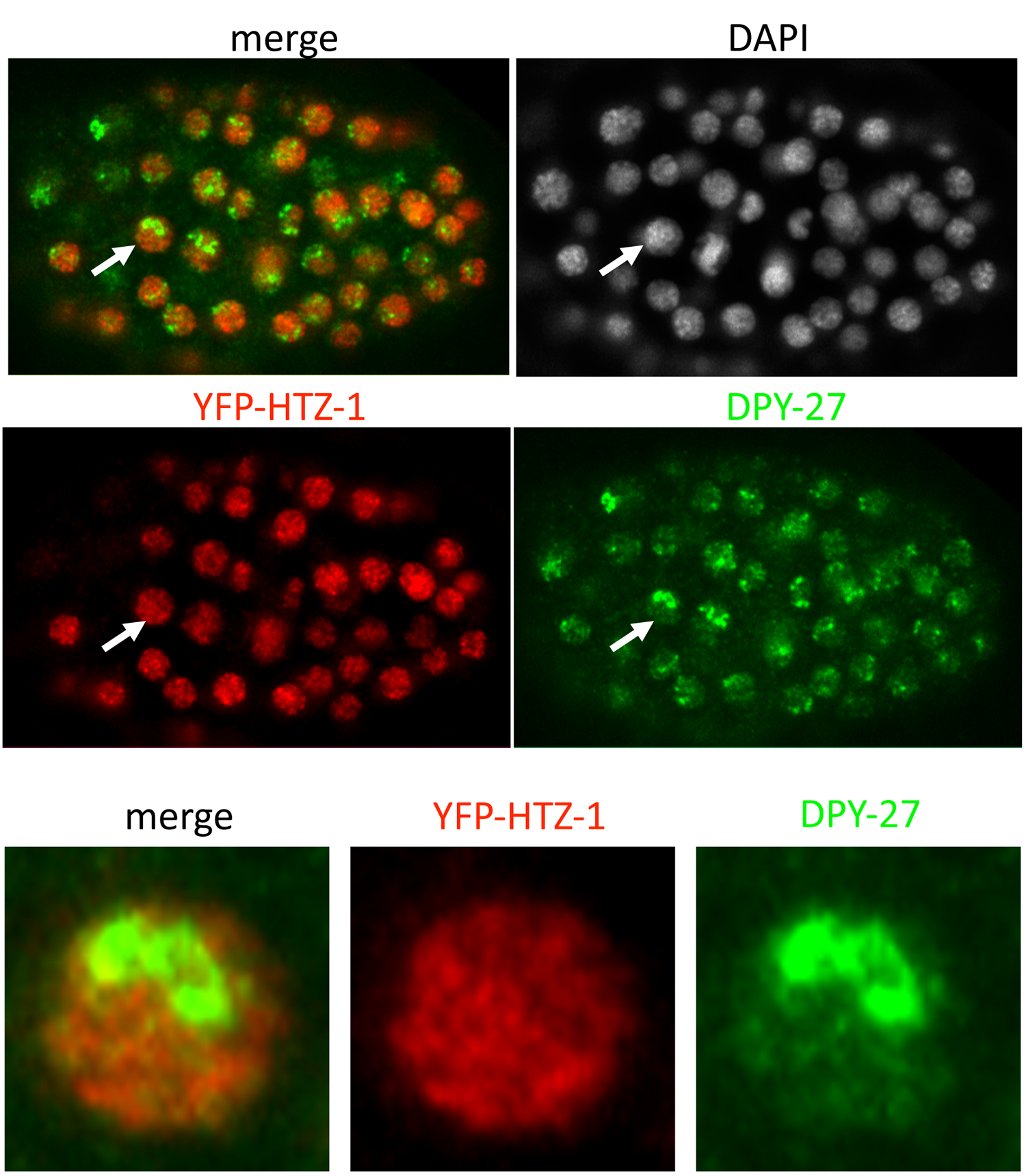

Supplement: Figure S2 — Transgenic YFP-HTZ-1 is also under-represented on the dosage compensated X chromosomes. Embryos after the onset of dosage compensation were stained with α-GFP antibodies to observe YFP-HTZ-1 localization (red), α-DPY-27 to mark the X chromosome (green) and DAPI (grayscale). Arrows in the top panels indicate enlarged nucleus shown below. YFP-HTZ-1 levels are reduced in the territory of the dosage compensated X chromosomes. (7.78 MB TIF) [file pgen.1000699.s002.tif]

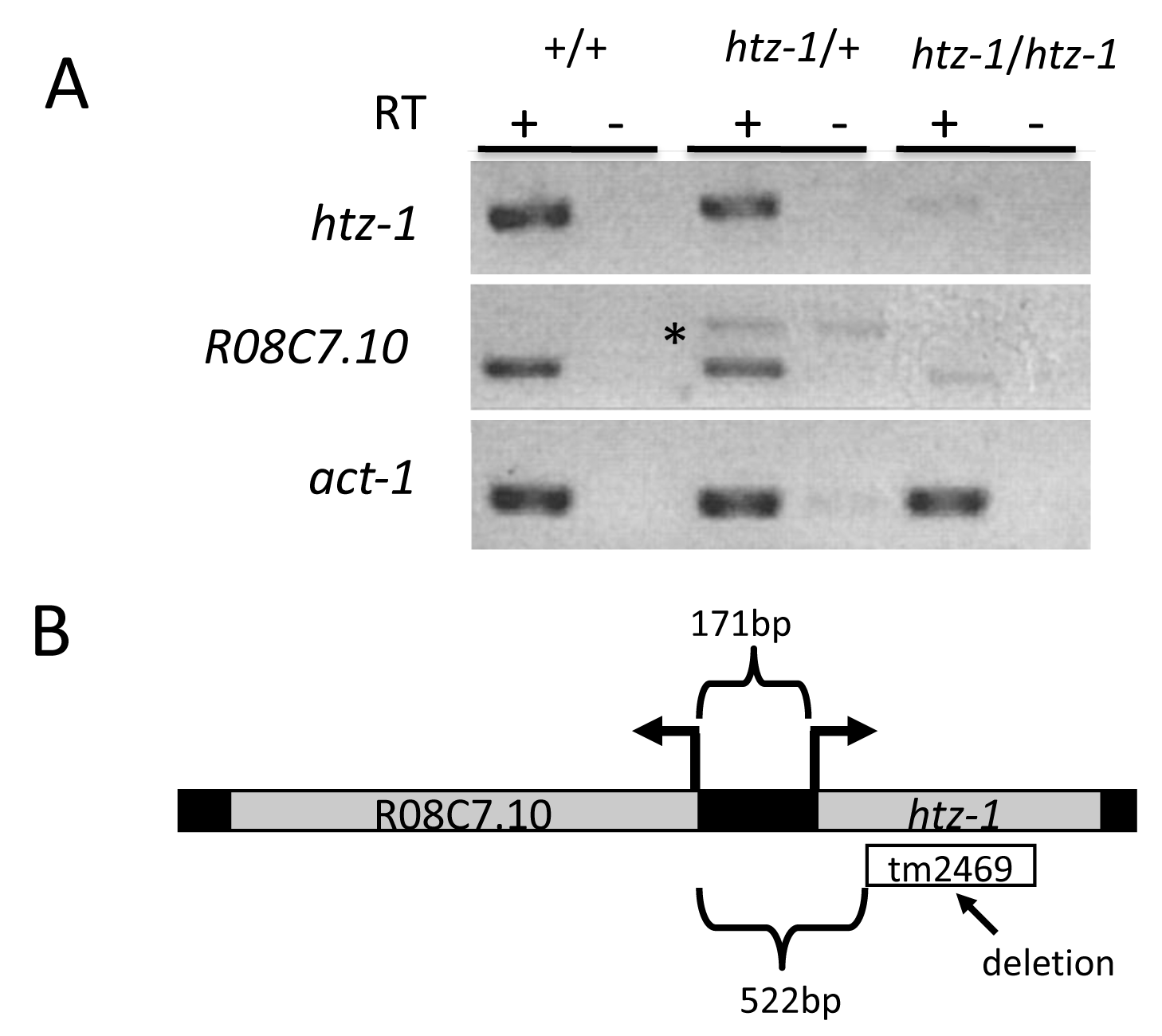

Supplement: Figure S3 — tm2469 deletion affects htz-1 and R08C7.10 expression. (A) Reverse-transcription polymerase chain reaction (RT-PCR) analysis of expression of htz-1, R08C7.10 and actin (control) in wild type (+/+), heterozygous (htz-1(tm2469)/nT1), or homozygous (htz-1(tm2469)) animals. Expression of both htz-1 and R08C7.10 is affected in homozygous animals. Contaminating amplification product from residual DNA in the RNA sample is indicated by a star. (B) Schematic showing relative positions of htz-1 and R08C7.10 on chromosome IV (not to scale). The tm2469 deletion removes most of the coding region of htz-1. In addition, it likely the affects the promoter or other cis control elements of the R08C7.10, a gene located only 522 base pairs away from the deletion. (5.72 MB TIF) [file pgen.1000699.s003.tif]

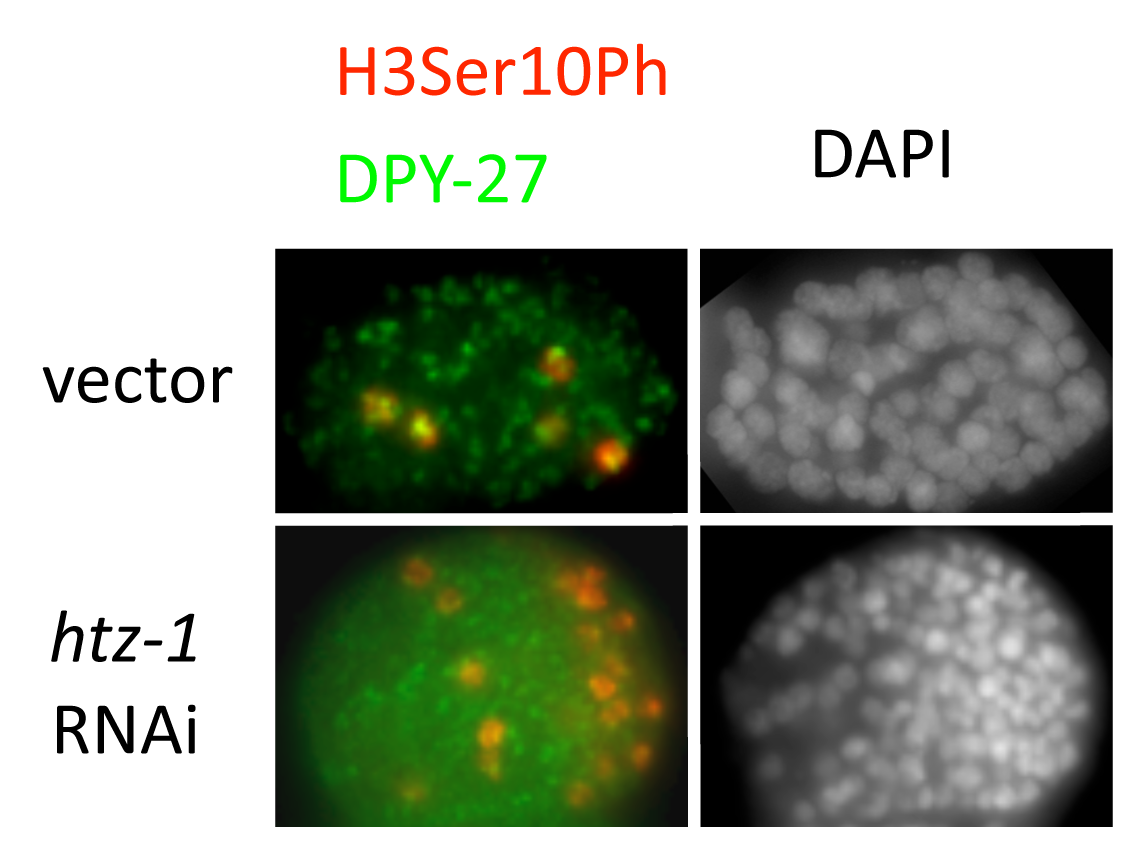

Supplement: Figure S4 — htz-1-depleted embryos also show compromised DCC localization. Vector and htz-1 RNAi embryos were stained with α-Phospho-H3 Ser10 (red) (to mark mitotic nuclei) and α-DPY-27 (green). After htz-1 RNAi, 16% of embryos with Phospho-H3 Ser10 staining (>50 cell stage) had diffuse nuclear DPY-27 localization (n = 372), as opposed to 2% in vector embryos (n = 314). (2.89 MB TIF) [file pgen.1000699.s004.tif]
