# Supplementary material for: Restricting Dosage Compensation Complex Binding to the X Chromosomes by H2A.Z/HTZ-1
Source: PLoS Genet. 2009 Oct 23;5(10):e1000699. doi: 10.1371/journal.pgen.1000699 (PMC2760203; doi:10.1371/journal.pgen.1000699)
Supplement: Table S1 — RNAi of the following genes did not result in significant (>10%) male rescue. (0.12 MB DOC) [file pgen.1000699.s005.doc]

**Table S1.** RNAi of the following genes did not result in significant (> 10%) male rescue.

| **locus** | **gene name** | **description** |
| --- | --- | --- |
| Y17G7B.2 | ash-2 | homolog of Drosophila Ash2, member of histone acetyltransferase complex |
| F25D7.3 | blmp-1 | zinc finger and SET domain containing protein |
| R10E11.1 | cbp-1 | homolog of mammalian CBP/p300 histone acetyltransferase |
| T14G8.1 | chd-3 | SNF2 family member, contain chromo domain |
| F20D12.1 | csr-1 | Argonaute protein required for chromosome segregation |
| ZK783.4 | flt-1 | PHD and bromo domain containing protein |
| M04B2.3 | gfl-1 | TFIIF transcription factor |
| C46A5.9 | hcf-1 | putative subunit of COMPASS histone methyltransferase complex |
| C08B11.2 | hda-2 | class I histone deacetylase |
| C10E2.3 | hda-4 | class II histone deacetylase |
| F22F1.1 | hil-3 | H1.3 histone |
| C18G1.5 | hil-4 | H1.4 histone |
| B0414.3 | hil-5 | H1.5 histone |
| F59A7.4 | hil-6 | H1.6 histone |
| C01B10.5 | hil-7 | H1.Q histone |
| M163.3 | his-24 | H1.1 histone |
| F45E1.6 | his-71 | H3.3 histone variant |
| Y49E10.6 | his-72 | H3.3 histone variant |
| K08H2.6 | hpl-1 | Heterochromatin protein 1 homolog |
| K01G5.2 | hpl-2 | Heterochromatin protein 1 homolog |
| F37A4.8 | isw-1 | homolog of chromatin remodeling ATPase ISW1 |
| F26F12.7 | let-418 | homolog of Mi-2/CHD3, subunit of NURD complex |
| F11A10.1 | lex-1 | similar to yeast Yta7p, contains bromo domain |
| F42A9.2 | lin-49 | zinc finger and bromo domain containing protein |
| F42A9.2 | lin-49 | zinc finger and bromo domain containing protein |
| T12F5.4 | lin-59 | SET domain containing protein |
| T08D10.2 | lsd-1 | homolog of histone demethylase LSD1 |
| C01G8.9 | lss-4 | ARID/BRIGHT DNA binding domain containing protein |
| M04B2.1 | mep-1/gei-2 | putative NURD/CHD complex subunit |
| R06A4.7 | mes-2 | Homolog of Enhancer of zeste, contains SET domain |
| F54C1.3 | mes-3 | member of the Polycomb-like repressive complex |
| Y2H9A.1 | mes-4 | SET domain containing protein, germline development |
| C09G4.5 | mes-6 | homolog of Drosophila extra sex combs, interacts with MES-2 |
| R05D3.11 | met-2 | H3K36 and H3K9 methylation |
| Y37D8A.9 | mrg-1 | homolog of mammalian MRG15, contains chromodomain |
| VC5.4 | mys-1 | MYST family histone acetyltransferase |
| K03D10.3 | mys-2 | MYST family histone acetyltransferase, similar to MOF |
| C34B7.4 | mys-4 | MYST family histone acetyltransferase |
| C26C6.1 | pbrm-1 | similar to human polybromo 1 |
| Y47G6A.6 | pcaf-1 | PCAF/GCN5-like histone acetyltransferase |
| F02E9.4 | pqn-28 | SIN3 homolog |
| C01G5.2 | prg-2 | Piwi subfamily Argonaute protein, germ-line stem cell division |
| F01G4.1 | psa-4 | SWI2/SNF2 ortholog |
| C47E12.4 | pyp-1 | similar to Drosophila NURF-38 and human PPA1 |
| K07A1.11 | rba-1 | nucleosome remodeling factor, subunit CAF1 |
| B0205.3 | rpn-10 | implicated in histone ubiquitination |
| ZK20.5 | rpn-12 | implicated in histone ubiquitination |
| T26A5.7 | set-1 | SET domain containing protein |
| F34D6.4 | set-11 | H3K9 methyltransferase homolog |
| K09F5.5 | set-12 | H3K9 methyltransferase homolog |
| R11E3.4 | set-15 | SET domain containing protein, germline development |
| T12D8.1 | set-16 | zinc finger and SET domain containing protein |
| T21B10.5 | set-17 | SET domain containing protein, similar to human PRDM11 and PRDM7 |
| W01C8.3 | set-19 | SET domain containing protein |
| C26E6.9 | set-2 | SET domain containing protein |
| Y32F6A.1 | set-22 | SET domain containing protein |
| Y41D4B.12 | set-23 | SET domain containing protein |
| C07A9.7 | set-3 | similar to human SMYD4, contains SET domain |
| C32D5.5 | set-4 | H4K20 methyltransferase homolog |
| C47E8.8 | set-5 | SET domain containing protein |
| C49F5.2 | set-6 | H3K9 methyltransferase homolog |
| F02D10.7 | set-8 | paralog of histone methyltransferase MES-4 |
| F15E6.1 | set-9 | PHD-zinc finger and SET domain containing protein |
| K12C11.2 | smo-1 | SUMO |
| R07E5.3 | snfc-5 | ortholog of SWI/SNF subunit SNF5 |
| C50E10.4 | sop-2 | SAM domain, member of Polycomb group |
| Y40B1B.6 | spr-5 | homolog of histone demethylase LSD1 |
| W04A8.7 | taf-1 | homolog of TAF250, histone acetyltransferase |
| C14B1.4 | tag-125 | homolog of histone methyltransferase subunit WDR5 |
| C01H6.7 | tag-298 | Bromo domain containing protein |
| ZK856.13 | tag-315 | RNA Pol III transcription factor TFIIIC |
| Y119C1B.8 | tag-332 | bromodomain containing protein |
| C47D12.1 | trr-1 | TRRAP-like histone acetyltransferase complex subunit homolog |
| D2013.9 | ttll-2/set-7 | putative tubulin polyaminoacid ligase |
| D2096.8 | D2096.8 | similar to nucleosome assembly protein NAP-1 |
| F32E10.2 | F32E10.2 | HP-1 like and chromo domain containing protein |
| F32E10.6 | F32E10.6 | chromo domain containing protein |
| F52B11.1 | F52B11.1 | PHD finger, SynMuv suppressor |
| F59E12.1 | F59E12.1 | bromodomain containing protein |
| H06O01.2 | H06O01.2 | putative chromodomain helicase, similar to human CHD1 |
| H20J04.2 | H20J04.2 | similar to chromatin remodeling complex WSTF-ISWI large subunit |
| K06A5.1 | K06A5.1 | chromo domain containing protein |
| K08F4.2 | K08F4.2 | SynMuv suppressor, involved in germline silencing |
| Y37D8A.11 | Y37D8A.11 | chromo domain containing protein |
| F54E12.2 | F54E12.2 | RNA Pol II termination factor TTF2-like |
| ZK1127.3 | ZK1127.3 | putative subunit of TPI60/NuA4 histone acetyltransferase complex |
| ZK1127.7 | ZK1127.7 | DNA gyrase/topoisomerase |
| T09A5.8 | T09A5.8 | chromo domain containing protein |
| T12E12.2 | T12E12.2 | chromo domain containing protein |
| T23B12.1 | T23B12.1 | Polycomb-like PHD zinc finger protein |
| Y57G11C.19 | Y57G11C.19 | chromo domain containing protein |
| C50A2.2 | C50A2.2 | chromo domain containing protein |
| CD4.7 | CD4.7 | putative SWR1/SCRAP complex member |
